# A Cytotoxic Heterodimeric Cyclic Diarylheptanoid with a Rearranged Benzene Ring from the Seagrass *Zostera marina*

**DOI:** 10.1021/acs.jnatprod.2c00796

**Published:** 2022-10-20

**Authors:** Laura Grauso, Yan Li, Silvia Scarpato, Nunzio Antonio Cacciola, Paola De Cicco, Christian Zidorn, Alfonso Mangoni

**Affiliations:** † Dipartimento di Agraria, Università degli Studi di Napoli Federico II, Via Università 100, 80055 Portici, Napoli, Italy; § Pharmazeutisches Institut, Abteilung Pharmazeutische Biologie, Christian-Albrechts-Universität zu Kiel, Gutenbergstraße 76, 24118 Kiel, Germany; ⊥ Dipartimento di Farmacia, Università degli Studi di Napoli Federico II, Via D. Montesano 49, 80131 Napoli, Italy; # Dipartimento di Medicina Veterinaria e Produzioni Animali, Università degli Studi di Napoli Federico II, Via F. Delpino, 80137 Napoli, Italy

## Abstract

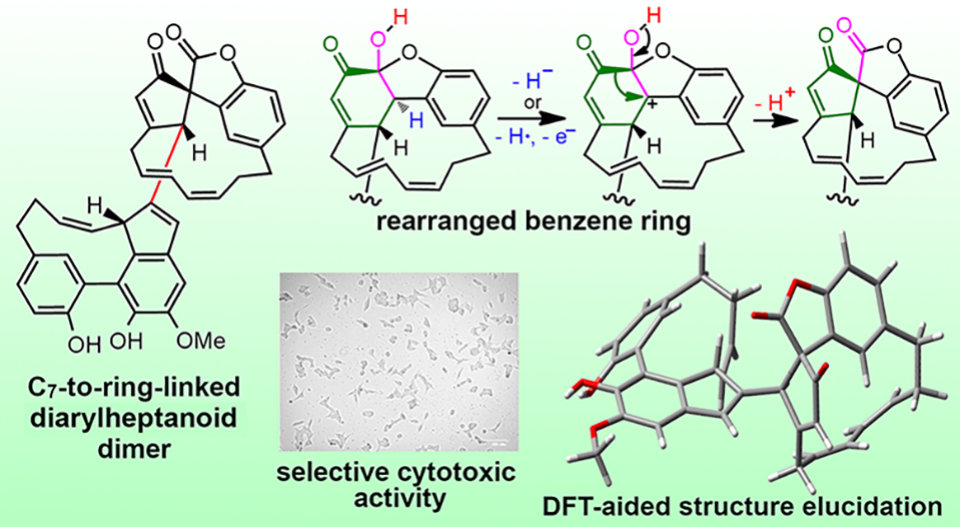

The widespread seagrass *Zostera marina* contains a new diarylheptanoid heterodimer, zosterabisphenone C (**1**), featuring an unprecedented rearrangement of one of its benzene rings to a cyclopentenecarbonyl unit. The planar structure and absolute configuration of zosterabisphenone C were elucidated by a combination of spectroscopic (MS, ECD, and low-temperature NMR) and computational (DFT-NMR and DFT-ECD) evidence. Consistent with the previously isolated zosterabisphenones, compound **1** was selectively cytotoxic against HCT 116 adenocarcinoma colon cancer cells, reducing their viability by 73% at 10 μM (IC_50_ of 7.6 ± 1.1 μM). The biosynthetic origin of zosterabisphenone C (**1**) from an oxidative rearrangement of zosterabisphenone A (**4**) is proposed.

Diarylheptanoids are natural products composed of two mono- or polyhydroxylated benzene rings joined by a functionalized seven-carbon chain. *Zostera marina* L. (Zosteraceae), a common and easily accessible seagrass that is widespread in the North Atlantic and North Pacific, has recently been shown to contain a rich family of cyclic diarylheptanoids with intriguing structural features.^1−4^ Among them are zosteraphenols A (**2**) and B (**3**) (Chart 1), tetracyclic diarylheptanoids showing coalescent NMR signals caused by the equilibrium with a minor rotamer with opposite axial chirality, and zosterabisphenones A (**4**) and B (**5**) (Chart 2), dimeric diarylheptanoids featuring unprecedented keto tautomers of catechol that are stable for steric reasons (for the complete set of diarylheptanoid structures found so far in *Z. marina*, see Chart S1).

**Chart 1:**
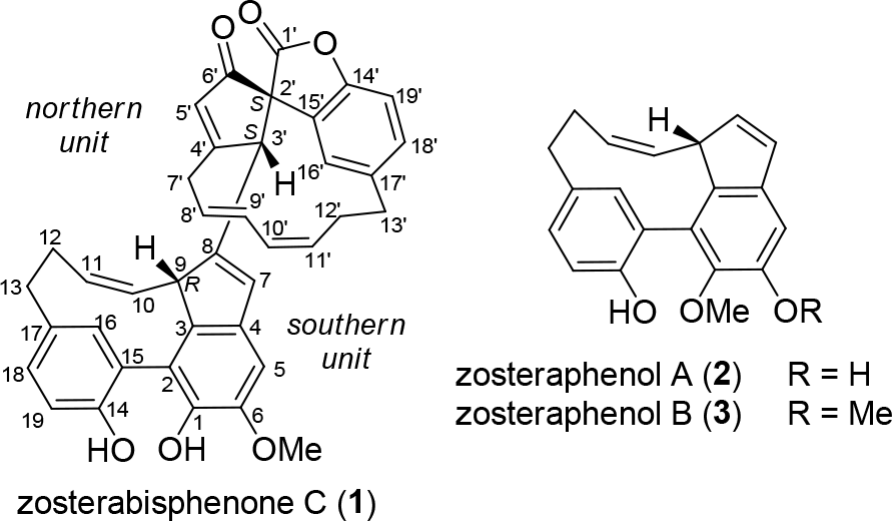


**Chart 2:**
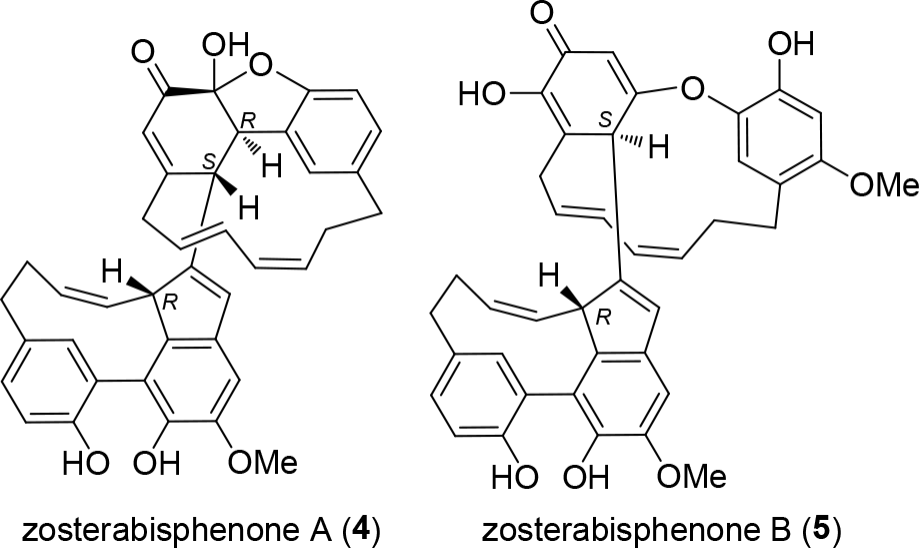


Further examination of the extracts of *Z. marina* revealed the presence of another dimeric diarylheptanoid, zosterabisphenone C (**1**). Zosterabisphenone C shares with zosterabisphenone A and B the unique dimeric diarylheptanoid structure resulting from the oxidative coupling of the central C_7_ chain of one unit with an aromatic ring of the other unit. In addition to this, zosterabisphenone C has another novel structural feature, namely, the rearrangement of one of its benzene rings to a cyclopentenecarbonyl unit. The isolation, elucidation of structure and absolute configuration, and evaluation of biological activity of zosterabisphenone C are reported here.

## Results and Discussion

Specimens of *Z. marina* (unrooted plants, freshly washed ashore and air-dried) were extracted with acetone at room temperature five times. The extract was subjected, in sequence, to SiO_2_ column chromatography (hexane/CH_2_Cl_2_/MeOH step gradient), Sephadex LH-20 chromatography (CH_2_Cl_2_/acetone at 85:15), and reversed-phase HPLC (MeOH/0.025% formic acid in H_2_O at 7:3) to give pure zosterabisphenone C (**1**, 10.3 mg).

**Figure 1:**
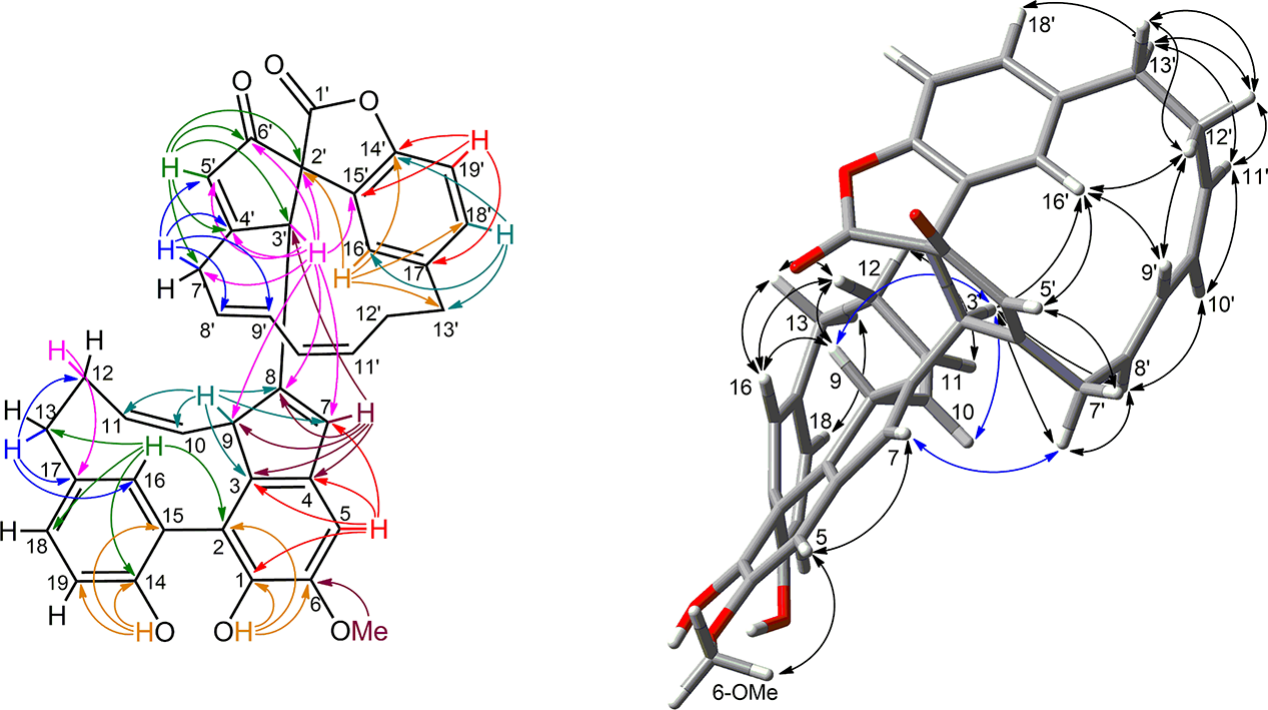
Most significant 2D-NMR data of zosterabisphenone C (**1**). Left: HMBC correlations; for clarity, arrows are color-coded according to the proton involved. Right: ROESY correlations, shown using the minimum energy conformation as determined by the DFT calculation.

The molecular formula of zosterabisphenone C (**1**) was determined to be C_39_H_32_O_6_ (24 unsaturations) from the [M + H]^+^ ion at *m*/*z* 597.2253. This formula was indicative of a dimeric diarylheptanoid and consistent with a dehydro derivative of zosterabisphenone A (**4**). The conformational equilibrium observed with zosteraphenols **2** and **3**(2) and zosterabisphenones **4** and **5**,^3^ leading to broad coalescent signals in NMR spectra recorded at room temperature, was also in effect for compound **1**. Therefore, all NMR spectra were recorded at 253 K, where all signals were sharp enough for structure elucidation. The combined analysis of COSY, HSQC, and HMBC NMR spectra showed that the southern diarylheptanoid unit of **1** was identical to that of zosterabisphenone A, and indeed, the relevant ^13^C NMR chemical shifts and ^1^H–^1^H coupling constants were all very similar to those reported for **4** (Table 1, Figure 1).^3^ In contrast, the ^1^H chemical shifts of many protons were remarkably shifted, suggesting large differences in the shielding/deshielding effects exerted by the northern unit.

**Table 1:** ^1^H and ^13^C NMR Data of Zosterabisphenone C (**1**) (700 MHz, 253 K, CDCl_3_)^a^

	zosterabisphenone C (**1**)					zosterabisphenone A (**4**)	
position	δ_C_, type		δ_H_ (*J* in Hz)	HMBC	ROESY	δ_C_, type	δ_H_ (*J* in Hz)
1	138.2, C		–	–		138.0, C	–
1-OH			6.71, s	1,2,6	—		6.74, s
2	122.2, C		–	–		122.4, C	–
3	140.4, C		–	–		139.7, C	–
4	137.3, C		–	–		137.3, C	–
5	103.4, CH		6.89, s	1,2,3,4,6,7	6-OMe,7	103.0, CH	6.80, s
6	145.8, C		–	–		145.9, C	–
6-OMe	56.2, CH_3_		3.95, s	6	5	56.2, CH_3_	3.96, s
7	130.1, CH		6.52, s	3,4,8,9,3′	5,7_pro*S*_^′^	128.8, CH	6.25, s
8	144.0, C		–	–		148.3, C	–
9	53.0, CH		4.58, br. d (11.1)	3,7,8,10,11	12_pro*R*_,16,3′	51.3, CH	5.01, br. d (11.2)
10	125.9, CH		4.41, t (11.1)	8,9,12	3′	127.4, CH	4.76, t (11.2)
11	135.0, CH		5.08, ddd (11.8, 11.1, 4.6)	9	12_pro*S*_	134.4, CH	5.55, ddd (11.8, 11.2, 4.8)
12	26.5, CH_2_	pro*R*	2.70, overlapped	10,11,13	9,12_pro*S*_,13_pro*S*_,16	27.6, CH_2_	3.05, dddd (13.6, 12.6, 11.8, 7.0)
		pro*S*	2.01, ddd (13.6, 6.1, 4.6)	10,11,13,17	11,12_pro*R*_,13_pro*R*_,13_pro*S*_		2.56, overlapped
13	34.0, CH_2_	pro*R*	2.50, ddd (14.4, 12.1, 6.1)	12,16,17	12_pro*S*_,13_pro*S*_,18	34.1, CH_2_	2.71, ddd (14.4, 12.6, 6.0)
		pro*S*	3.30, dd (14.4, 6.7)	12,16,17	12_pro*R*_,12_pro*S*_,13_pro*R*_,16		3.39, dd (14.4, 7.0)
14	151.8, C		–	–		152.0, C	–
14-OH	–		7.51, s	14,15,19		–	7.57, s
15	122.8, C		–	–		122.7, C	–
16	135.1, CH		7.56, br. s	2,13,14,18	9,12_pro*R*_,13_pro*S*_	135.1, CH	7.68, br. s
17	132.1, C		–	–		131.9, C	–
18	129.5, CH		6.80, br. d (8.0)	13,14,16	13_pro*R*_	129.7, CH	6.91, br. d (8.0)
19	116.4, CH		6.78, d (8.0)	14,15,17	–	116.7, CH	6.86, d (8.0)
1′	171.8, C		–	–		103.9, C	
2′	65.4, C		–	–		50.2, CH	3.85, s
3′	54.7, CH		3.68, s	7,8,9,2′,4′,5′,6′,7′,15′	9,10,7_pro*S*_^′^,8′,16′	46.1, CH	3.56, s
4′	181.2, C		–	–		163.3, C	–
5′	127.5, CH		6.43, s	2′,3′,4′,6′,7′	7_pro*R*_^′^,16′	122.6, CH	6.18, s
6′	200.1, C		–	–		189.1, C	–
7′	31.2, CH_2_	pro*R*	3.51, dd (14.4, 6.5)	3′,4′,5′,8′,9′	5′,7_pro*S*_^′^	38.5, CH_2_	2.97, dd (12.4, 7.4)
		pro*S*	3.00, overlapped	4′,5′,8′,9′	7,3′,7_pro*R*_^′^,8′		2.33, overlapped
8′	125.5, CH		5.53, ddd (15.7, 8.3, 6.5)	7′,10′	3′,7_pro*S*_^′^,10′	124.5, CH	5.08, ddd (15.3, 8.0, 8.0)
9′	133.5, CH		6.72, dd (15.7, 10.8)	7′,10′,11′	12_pro*R*_^′^,16′	128.1, CH	5.89, dd (15.3, 10.6)
10′	127.9, CH		5.89, t (10.8)	8′,9′,12′	8′,11′	129.2, CH	5.65, t (10.6)
11′	131.0, CH		5.34, ddd (11.8, 10.8, 4.8)	9′,12′	10′,12_pro*S*_^′^,13_pro*R*_^′^	129.4, CH	5.22, ddd (11.5, 10.6, 5.3)
12′	24.3, CH_2_	pro*R*	2.95, dddd (14.2, 13.2, 11.8, 2.9)	13′	12_pro*S*_^′^,16′	29.4, CH_2_	2.31, overlapped
		pro*S*	2.33, m	13′	11′,12_pro*R*_^′^,13_pro*R*_^′^,13_pro*S*_^′^		2.53, dq (3.3, 12.2)
13′	31.7, CH_2_	pro*R*	2.66, ddd (16.7, 13.2, 2.9)	11′,12′,16′,17′,18′	11′, 12_pro*S*_^′^,13_pro*S*_^′^,18′	33.6, CH_2_	2.98, overlapped
		pro*S*	3.02, overlapped	11′	12_pro*S*_^′^,13_pro*R*_^′^,18′		2.34, overlapped
14′	150.3, C		–	–		154.9, C	–
15′	128.3, C		–	–		127.8, C	–
16′	119.4, CH		7.06, overlapped	2′,13′,14′,17′,18′	3′,5′,9′,12_pro*R*_^′^	123.4, CH	6.85, br. s
17′	135.8, C		–	–		133.9, C	–
18′	130.2, CH		7.06, overlapped	13′,14′,16′,17′	13_pro*R*_^′^,13_pro*S*_^′^	130.5, CH	6.70, br. d (8.0)
19′	110.1, CH		7.01, d (8.6)	14′,15′,17′		109.0, CH	6.56, d (8.0)

a Reported^3^ NMR data of zosterabisphenone A (**4**) are also shown for comparison.

As for the northern diarylheptanoid unit, further examination of NMR data showed the presence of a 1,2,4-trisubstituted benzene ring and of a hepta-2,4-diene-1,7-diyl chain like in **1**. However, the second benzene ring of the northern diarylheptanoid unit was shown to be rearranged to a five-membered ring when considering the following HMBC information. The coupling of H-16′ with the quaternary sp^3^ carbon atom C-2′ established the C-15′/C-2′ bond and the coupling of the methine proton H-3′ with C-2′ and C-15′, the C-2′/C-3′ bond. The connection of the enone system C-4′/C-5′/C-6′ with C-3′ and C-7′ was demonstrated by the allylic couplings of H-5′ with H-3′ and H-7′_pro*S*_ observed in the COSY spectrum and confirmed by many HMBC cross peaks (Figure 1). The C-2′/C-6′ bond, closing a five-membered ring, was demonstrated by the HMBC correlations of H-5′ with C-2′ and H-3′ with C-6′. The last unassigned carbon atom in the ^13^C spectrum, an ester carbonyl resonating at δ 171.8, showed no HMBC correlation but, at this stage, could only be linked to C-2′ and the oxygen atom at C-14′ to close a further five-membered lactone ring, spiro-fused to the carbocyclic five-membered ring. The C-8/C-3′ linkage between the two diarylheptanoid units was demonstrated by the HMBC correlations of H-3′ with C-7′, C-8′, and C-9′ and that of H-7 with C-3′.

The relative configuration of C-2′ and C-3′, the two stereocenters in the northern unit, was established from the ROESY correlations of H-3′ with H-8′ and H-16′, showing H-3′ pointing inward of the macrocycle; the conformation of the C_7_ bridge, determined on account of the strong ROESY cross-peaks observed between H-9′, H-12′_pro*R*_, and H-16′, was shown to be basically rigid by a preliminary molecular modeling/density functional theory (DFT) study, reported in detail in the Supporting Information. The configuration and conformation of the two diarylheptanoid units was additionally supported by the DFT prediction of ^1^H–^1^H scalar couplings,^5^ showing excellent agreement with the multiplicity of signals observed in the ^1^H NMR spectrum (Table S5).

The relative configuration of the two diarylheptanoid units was determined computationally by a DFT study. The study was performed using the Gaussian 16 program and a computational protocol similar to the one used in the previous work^3^ and is described in detail in the Supporting Information.

Discrimination between the two possible diastereomers (9*R*,2′*S*,3′*S*)-**1** (just **1** in the following text) and (9*S*,2′*S*,3′*S*)-**1** (*epi*-**1** in the following text) was based on the DFT prediction of ^1^H and ^13^C NMR chemical shifts. Models of the two diastereomers were generated and optimized by DFT. Rotation about the bond C-8/C-3′, the only unknown degree of freedom in the molecule, was scanned, and the resulting structures were reoptimized. This resulted in one low-energy conformer for **1** and two low-energy conformers for *epi*-**1** (Figure S4). Finally, ^1^H and ^13^C NMR chemical shifts were calculated at the PBE0/6-311+G(2d,p)/PCM level of theory using the scaling factors proposed by the Tantillo group^6^ (Tables S3 and S4, Figure S3).

Predicted chemical shifts of **1** were in much better agreement with the experiment (RMSD of 1.86 ppm for ^13^C and 0.134 ppm for ^1^H) than those of *epi*-**1** (RMSD of 2.10 ppm for ^13^C and 0.168 ppm for ^1^H) (Figure S3). Moreover, the DP4+ probability^7^ of structure **1** compared to *epi*-**1** was 100.00%. Structure **1** also nicely explained the absence of an HMBC correlation between C-1′ and H-3′ (the only proton within three bonds from C-1′), because the 93° value measured for the dihedral angle C-1′/C-2′/C-3′/H-3′ implied the coupling between the two nuclei was close to zero.

The absolute configuration of **1** was assigned on the basis of its electronic circular dichroism (ECD) spectrum. The ECD transitions of the 9*R*,2′*S*,3′*S* stereoisomer of **1**, the enantiomer randomly chosen for calculations, were calculated at the ωB97XD/6-31+G(d,p)/PCM level of theory, and a predicted ECD spectrum was generated with the aid of the SpecDis program^8^ (σ of 0.30 eV, UV correction of +15 nm). This predicted ECD spectrum was in good agreement with the experimental spectrum (Figure S12), thus determining the absolute configuration of zosterabisphenone C (**1**) to be (9*R*,2′*S*,3′*S*).

**Chart 3:**
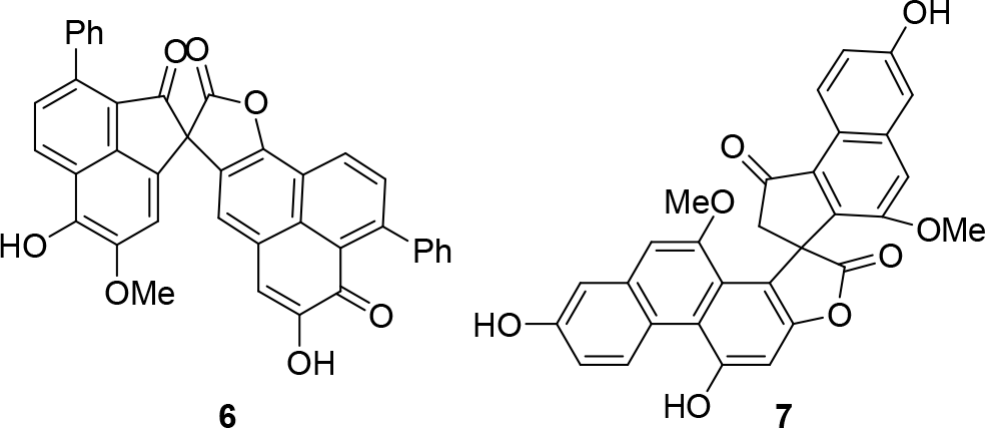


Zosterabisphenone C (**1**) is characterized by the rearrangement of one benzene ring to a carboxycyclopentenone, observed here for the first time in a diarylheptanoid. Only two similar, but different, spirolactone systems have been reported so far as natural products, namely, lachnanthospirone (**6**)^9^ and dendrochrysanene (**7**)^10^ (Chart 3), and both are rearranged products from symmetric aromatic dimers. A total synthesis of dendrochrysanene has been reported from a phenolic precursor,^11^ involving FeCl_3_-promoted one-step dimerization and framework rearrangement of the dimer.^12^

**Scheme 1:**
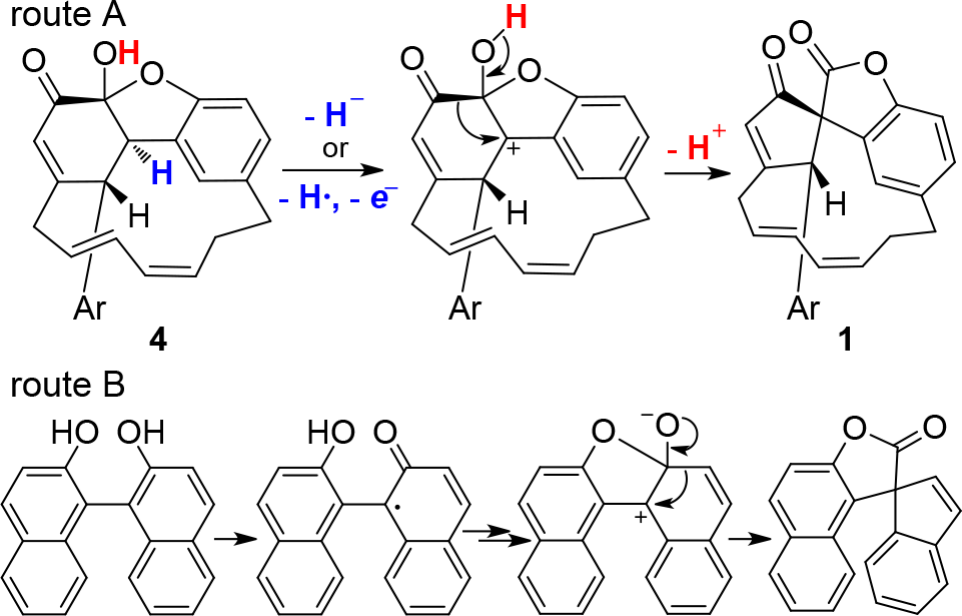
Route A: Putative Mechanism of Conversion of Zosterabisphenone A (**4**) into Zosterabisphenone C (**1**); Route B: Mechanism Proposed in Ref (12) for the FeCl_3_-Promoted Rearrangement of 2,2′-Dinaphthol Ar is the southern diarylheptanoid unit in Route A.

Similarly, compound **1** can reasonably be derived from the oxidative rearrangement of zosterabisphenone A (**4**). Scheme 1 shows a plausible mechanism for this rearrangement (route A), partly based on the mechanism proposed in ref (12) for the rearrangement of 2,2′-binaphthol derivatives (route B). Hydride abstraction at C-2′ by an oxidizing agent (possibly as abstraction of the benzylic hydrogen followed by one-electron oxidation) induces migration of the carbonyl group from C-1′ to C-2′, thus creating the spiro quaternary carbon atom and the ester function. The stereochemical relationship between zosterabisphenones A and B is fully consistent with the hypothesized suprafacial 1,2-shift.

Even though this mechanism is closely related to that proposed for the rearrangement of the naphthol dimer,^12^ it shows some important differences: (i) route B only works with condensed biaryls; in contrast, route A occurs with a (modified) biphenyl; (ii) in route A, the cleavage of a C–H bond must occur because the starting compound is the keto tautomer of a catechol; (iii) the hemiacetal function that is formed in route B is preformed in route A. With the available data, we cannot predict whether route A is amenable to be developed into a synthetically useful reaction or can be effective only with a highly sterically strained substrate such as zosterabisphenone A (**4**).

In a previous paper,^3^ zosterabisphenone B (**5**) was shown to be cytotoxic against the colorectal human cancer cell line HCT 116 (97% reduction of viability at 10 μM with a nice concentration–response curve and IC_50_ of 3.6 μM), selectively over the hepatic cell line Hep G2; in contrast, zosterabisphenone A (**4**) was only weakly active on HCT 116 cells (23% inhibition) at the highest concentration tested, 10 μM (Table S7).

**Figure 2:**
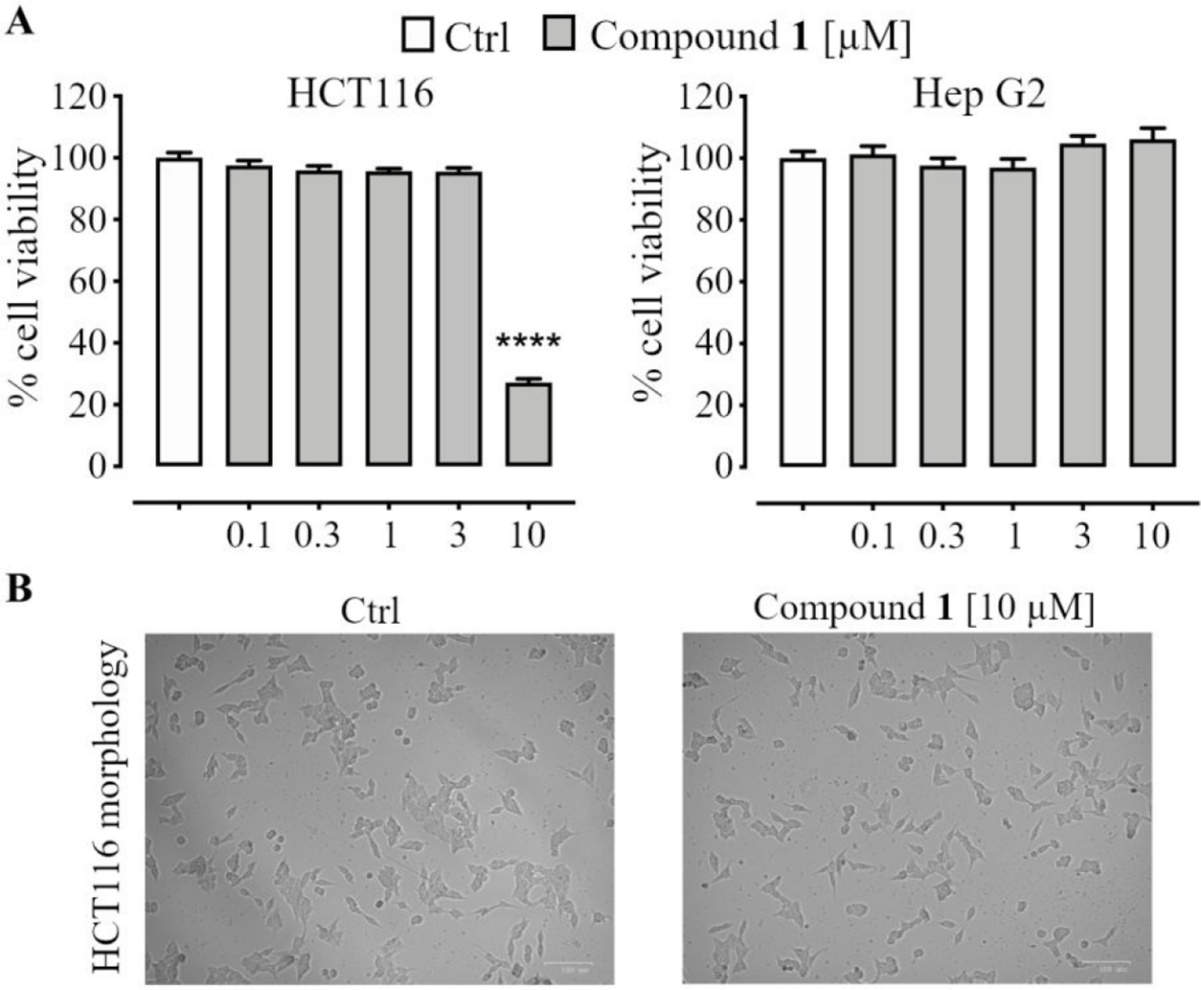
(A) Effect of zosterabisphenone C (**1**) (0.1–10 μM, 48 h exposure) on the viability of HCT 116 and Hep G2 cells, as determined by the MTT assay; ****, *p* < 0.0001 vs control (Ctrl). (B)Morphological changes in HCT 116 colon cancer cells after 48 h of treatment with 10 μM zosterabisphenone C (**1**). Left panel, control cells (treated with 0.1% ethanol); right panel, cells treated with 10 μM zosterabisphenone C (**1**). Cells were visualized under a phase-contrast microscope (magnification, 100×).

The cytotoxic effect of zosterabisphenone C (**1**) was studied using the same two cell lines and was found to be in between those of compounds **5** and **4**. As shown in Figure 2A, treatment with compound **1** significantly reduced the viability of HCT 116 cells (inhibition as high as 73%), but this effect was only observed at the highest concentration tested (10 μM). A further experiment with additional concentration levels (data not shown) determined the IC_50_ of compound **1** as 7.6 ± 1.1 μM. Selectivity was maintained, as compound **1** did not reduce Hep G2 cell viability at any of the concentrations tested. Additional information was provided by phase-contrast microscopy of untreated and zosterabisphenone-C-treated HCT 116 cells (Figure 2B). Exposure of HCT 116 cells to 10 μM of zosterabisphenone C (**1**) for 48 h resulted in morphological cellular changes indicative of cell death and growth inhibition. Specifically, treatment with zosterabisphenone C significantly impaired the spreading and elongation of HCT 116 cells; the cells showed a long, thin, spindle-shaped form with boundaries resembling those of loosely adherent cells.

Washed-up biomass of *Z. marina* is freely available in tons on the shores of the North Atlantic and North Pacific; in addition, seeds of *Z. marina* have recently been proposed as a new food, and an experimental cultivation of *Z. marina* has been established;^13^ so, the future availability of large amounts of byproduct biomass of *Z. marina* is a realistic possibility. Owing to their consistent cytotoxic activity and the wide distribution and abundance of their biological source, zosterabisphenones have the potential to be exploited as antitumor leads for human and/or veterinary uses. More detailed studies on the activity and the mechanism of action of zosterabisphenones and on the pathways involved are in progress and will be reported in due course.

## Experimental Section

### General Experimental Procedures

Optical rotations were measured on a Jasco P-200 polarimeter at 589 nm using a 10 cm cell. UV spectra were recorded on a Jasco V-530 spectrophotometer. ECD spectra were recorded on a Jasco 715 spectropolarimeter. IR spectra were recorded on a PerkinElmer Spectrum 100 FT-IR spectrometer. NMR spectra were recorded on a 700 MHz Bruker Avance Neo spectrometer equipped with a cold probe and a BCU-II variable temperature unit. Chemical shifts were referenced to the solvent peaks at δ_H_ 7.26 and δ_C_ 77.0 for CDCl_3_. High-resolution LC-ESI mass experiments were performed on a Thermo LTQ Orbitrap XL mass spectrometer coupled to a Thermo Ultimate 3000 UPLC system.

### Extraction and Isolation

Whole plants of *Zostera marina* L. were collected at the coast close to the Olympiazentrum Schilksee, Kiel, Schleswig-Holstein, Germany, in December 2018 (coordinates: N 54°25′39.0′′, E 10°10′17.5′′; alt.: 0 m). The collected plants were unrooted plants freshly washed ashore. A voucher specimen is preserved in the Herbarium of the Institute of Botany, Kiel University (voucher code: YL-20181222A-1; KIEL0005004).

Air-dried, ground whole plants of *Z. marina* (1.75 kg) were extracted with acetone at room temperature (five times, 5.5 L each), yielding 18.3 g of residue after evaporation of the solvent *in vacuo*. The extract was subjected to silica gel column chromatography on a 7.5 × 50 cm column, eluted in sequence with hexane/CH_2_Cl_2_ (7:3), hexane/CH_2_Cl_2_ (5:5), CH_2_Cl_2_/acetone (7:3), CH_2_Cl_2_/acetone (5:5), and acetone (1 L of each mixture) to yield 24 subfractions.

Fraction N (1.25 g), containing zosterabisphenones C (**1**), was further separated by medium pressure silica gel column chromatography, performed using a PrepChrom C-700 system (Büchi) with a silica gel column (Sepacore Silica 40–63 μm, 80 g, 194 × 31 mm) at a flow rate of 20.0 mL/min. The employed linear gradients were 0 min, hexane; 30 min, hexane/CH_2_Cl_2_ (8:2); 80 min, hexane/CH_2_Cl_2_ (1:1); 81 min, CH_2_Cl_2_; 120 min, CH_2_Cl_2_/MeOH (8:2); run time of 2 h, yielding a total of 15 fractions. Fraction N11 (114 mg, eluting with 68% hexane and 32% CH_2_Cl_2_) contained compound **1**.

Fractions N11 was further purified by Sephadex LH-20 column chromatography (2 × 100 cm) using CH_2_Cl_2_/acetone (85:15) as eluants. Partially purified compound **1** (27.5 mg) was finally purified by semipreparative reversed-phase HPLC using a Waters e2695 instrument and a Nucleodur C_8_ column (1 × 25 cm), flow rate of 2 mL/min, UV detection at 210 and 254 nm, and mixtures of MeOH and 0.025% formic acid in H_2_O as the mobile phase in isocratic separation. Pure zosterabisphenone C (**1**, 10.3 mg) was eluted isocratically with 75% MeOH (*t*_R_ of 40–46 min).

#### Zosterabisphenone C (**1**)

[α]_D_^25^ −29 (*c* 0.01, MeCN); UV/vis (MeCN): λ_max_ (log ε) 300 (3.95), 210 nm (4.53); ECD (MeCN): λ_max_ (Δε) 312 (+8.3), 276 (−6.0), 235 (−43.6), 217 (+60.5), 197 nm (+22.3); IR (solid film) ν_max_ 3361, 2924, 1807, 1714, 1608, 1489, 1458, 1421, 1321, 1255, 1230, 972, 737 cm^–1^; ^1^H NMR and ^13^C NMR data, see Table 1; HRMS (ESI/Orbitrap) *m*/*z* 597.2253 [M + H]^+^ (calcd for C_39_H_33_O_6_^+^, 597.2272), 614.2527 [M + NH_4_]^+^ (calcd for C_39_H_36_O_6_N^+^, 614.2537), 619.2081 [M + Na]^+^ (calcd for C_39_H_34_O_6_Na^+^, 619.2091), 629.2523 [M + MeOH + H]^+^ (calcd for C_40_H_37_O_7_^+^, 629.2534).

### General Computational Methods

A conformational search was performed using molecular dynamics (MD) with the INSIGHT II/Discover package (BIOVIA). All the MD simulations were performed at 2000 K to allow possible slow conformational changes to occur in the short duration of the simulation,^3^ constraining the geometry of the double bonds to prevent *cis*/*trans* isomerization. The effect of the solvent (CHCl_3_) was approximated using a dielectric constant of 4.81. The search protocol involved a 10 ns MD simulation in the CFF91 force field. The coordinates were saved every 50 ps and subsequently minimized in the same force field, giving 200 minimized structures, which were used as input for the subsequent quantum-mechanical calculations.

DFT calculations were performed using the program Gaussian 16 (Revision C.01, Gaussian Inc.), using the B3LYP/6-31+G(d,p) level of theory for structure optimization, the Gauge Invariant Atomic Orbitals (GIAO) method^8^ at the PBE0/6-311+G(2d,p) level of theory and the PCM solvent model for the prediction of NMR chemical shifts, and the time-dependent DFT (TDDFT) method at the ωB97XD/6-31+G(d,p) level of theory and the PCM solvent model for the ECD prediction. Proton–proton NMR scalar couplings were calculated according to the suggestions of Bally and Rablen:^5^ calculations were performed at the B3LYP/6-31G(d,p) level of theory *in vacuo*, and only the Fermi contact terms were calculated, which were then scaled by a factor of 0.9117.

The NMR isotopic shielding computed for each H or C nucleus was converted into a chemical shift using the scaling factors proposed by the Tantillo group^6^ for the level of theory used (^1^H: slope −1.0958, intercept 31.7532; ^13^C: slope −1.0533, intercept: 187.3123). When more than one conformer was significantly populated, the weighted mean of the chemical shifts and scalar couplings of the individual conformers were calculated using Boltzmann statistics (*T* = 253 K).

The predicted ECD curves were obtained using the program SpecDis v. 1.71,^8^ adjusting the parameters σ and UV shift for the best fit between the predicted and experimental spectra. When more than one conformer was significantly populated, the weighted mean of the ECD curves of the individual conformers was calculated using Boltzmann statistics (*T* = 298 K).

### Cell Cultures

Human colorectal (HCT 116) and hepatocellular (Hep G2) carcinoma cell lines were purchased from the American Type Culture Collection (ATCC). HCT 116 cells were cultured in McCoy’s 5° medium (catalog number 10-050-CV, Corning) while Hep G2 cells were maintained in Minimum Essential Medium (MEM, catalog number AL047, Microgem). The cells were supplemented with 10% FBS (catalog number 1027-106, Gibco), 100 U/mL penicillin, and 100 μg/mL streptomycin (catalog number ECB3001, Euroclone). Cells were cultured in a humidified incubator at 37 °C with 5% CO_2_.

### Cell Viability Assay (MTT Assay)

The MTT [3-(4,5-dimethylthiazol-2-yl)-2, 5-diphenyltetrazolium bromide] assay was used to assess cell viability, as described previously.^3^ Briefly, HCT 116 cells (5× 10^3^ cells/well) and Hep G2 cells (1× 10^4^ cells/well) were seeded in 96-well culture plates for 24 h in medium containing 10% FBS. Cells were then treated with or without compound **1** at concentrations of 0–10 μM for 48 h in medium containing 1% FBS, and 20% DMSO was used as the positive control. At the end of the treatment period, an MTT stock solution (250 μg/mL) was added to each well. After an incubation period of 1 h at 37 °C, the intracellular formazan crystals were dissolved with DMSO and the absorbance of the solution was measured at 570 nm using a microplate reader (Cytation 3, BioTek Instruments, Inc.). The morphological changes of the cells were recorded with the ZOE Fluorescent Cell Imager (Bio-Rad)

### Statistical Analysis

Statistical analysis, performed using GraphPad Prism 7.0, was determined by two-way analysis of variance (ANOVA) followed by a Tukey-Kramer multiple comparisons test. *P* < 0.05 was considered significant. Data were expressed as the mean ± mean standard error (SEM) of *n* experiments.

## Supporting Information Available

Additional computational details with additional figures and tables; full results of assays; MS, IR, NMR, UV, and ECD spectra of **1** (PDF)
