# Supplementary material for: A Cytotoxic Heterodimeric Cyclic Diarylheptanoid with a Rearranged Benzene Ring from the Seagrass Zostera marina
Source: J Nat Prod. 2022 Oct 20;85(10):2468–73. doi: 10.1021/acs.jnatprod.2c00796 (PMC9623580; doi:10.1021/acs.jnatprod.2c00796)
Supplement: Supplementary file 1 — np2c00796_si_001.pdf [file np2c00796_si_001.pdf]

Supporting Information for

A Cytotoxic Heterodimeric Cyclic Diarylheptanoid with a Rearranged Benzene Ring from the Seagrass *Zostera marina*

Laura Grauso,[§,‡] Yan Li,[†,‡] Silvia Scarpato,[⊥] Nunzio Antonio Cacciola,[#] Paola De Cicco,[⊥,#] Christian Zidorn,[†] and Alfonso Mangoni*[,⊥]

[§] Dipartimento di Agraria, Università degli Studi di Napoli Federico II, Via Università 100, 80055 Portici (NA), Italy
[†] Pharmazeutisches Institut, Abteilung Pharmazeutische Biologie, Christian-Albrechts-Universität zu Kiel, Gutenbergstraße 76, 24118, Kiel, Germany
[⊥] Dipartimento di Farmacia, Università degli Studi di Napoli Federico II, Via D. Montesano 49, 80131 Napoli, Italy
[#] Dipartimento di Medicina Veterinaria e Produzioni Animali, Università degli Studi di Napoli Federico II, Via F. Delpino, 80137 Napoli, Italy

Table of Contents

**Chart S1**	Diarylheptanoids found in *Zostera marina*	2
**Computational details** (including **Figures S1-S2**)		3
**Figure S3**	Deviations of calculated ^{13}C and ^{1}H NMR chemical shifts of zosterabisphenone C (**1**) and its C-9 epimer *epi*-**1**	5
**Figure S4**	High-resolution ESI mass spectrum of zosterabisphenone C (**1**)	6
**Figure S5**	^{1}H NMR spectrum of zosterabisphenone C (**1**) recorded at 298 K and 253 K (700 MHz, $CDCl_3$)	7
**Figure S6**	^{13}C NMR spectrum of zosterabisphenone C (**1**) recorded at 253 K (175 MHz, $CDCl_3$)	8
**Figure S7**	HSQC spectrum of zosterabisphenone C (**1**) recorded at 253 K (700 MHz, $CDCl_3$)	9
**Figure S8**	HMBC spectrum of zosterabisphenone C (**1**) recorded at 253 K (700 MHz, $CDCl_3$)	10
**Figure S9**	Expansion of the HMBC spectrum of zosterabisphenone C (**1**) recorded at 253 K (700 MHz, $CDCl_3$)	11
**Figure S10**	COSY spectrum of zosterabisphenone C (**1**) recorded at 253 K (700 MHz, $CDCl_3$)	12
**Figure S11**	ROESY spectrum of zosterabisphenone C (**1**) recorded at 253 K (700 MHz, $CDCl_3$)	13
**Figure S12**	Calculated and experimental UV and ECD spectra of zosterabisphenone C (**1**) in acetonitrile	14
**Figure S13**	IR spectrum of zosterabisphenone C (**1**) (solid film)	15
**Table S1**	Cartesian coordinates of the lowest-energy conformer of zosterabisphenone C (**1**)	16
**Table S2**	Cartesian coordinates of the lowest-energy conformers of the C-9 epimer of zosterabisphenone C (*epi*-**1**)	17
**Table S3**	Experimental and predicted chemical shifts of zosterabisphenone C (**1**)	18
**Table S4**	Experimental and predicted chemical shifts of the two conformers of the C-9 epimer of zosterabisphenone C (*epi*-**1**)	19
**Table S5**	Experimental multiplicity of ^{1}H NMR signals and predicted ^{1}H-^{1}H *J* couplings of zosterabisphenone C (**1**)	20
**Table S6**	Rotatory strengths (length formalism) calculated for zosterabisphenone C (**1**)	21
**Table S7**	Effects of zosterabisphenone A (**4**), B (**5**), and C (**1**) on the viability of HCT116 and Hep G2 cells after 48 hours of treatment	22
**References**		22

Chart S1. Diarylheptanoid found in *Zostera marina*

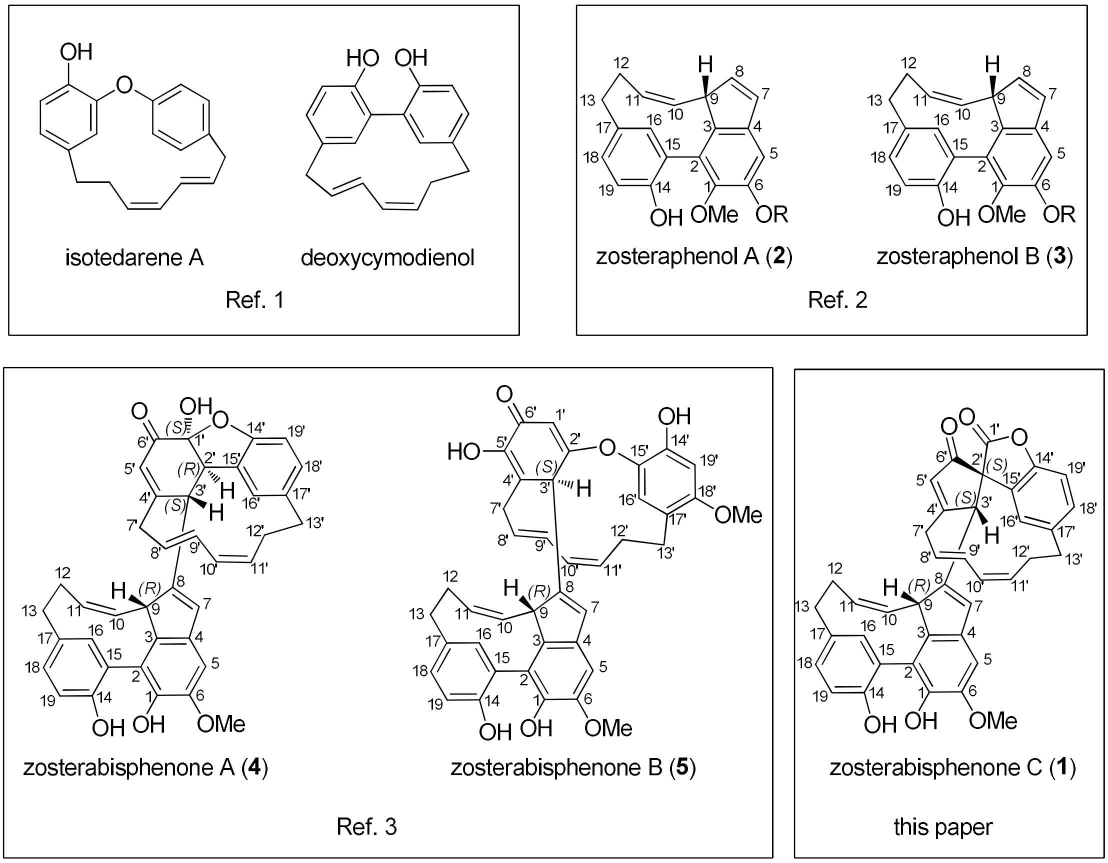

Additional Computational Details

A simplified model (**1n**) of the northern diarylheptanoid unit of zosterabisphenone C, with the southern unit replaced by a phenyl ring and with the relative configuration at C-2', and C-3' as determined by NMR studies (the 2'*S*,3'*S* enantiomer was tentatively used, Figure S1) was subjected to molecular dynamics (MD) conformational search. The MD simulation was set at 2000 K to allow possible slow conformational changes to occur in the 10-ns duration of the simulation.[3] This identified 5 unique conformers in a range of 6.93 kcal/mol, whose geometry was optimized by DFT at the B3LYP/6-31+G(d,p) level. The lowest-energy conformer was in full agreement with the ROESY data (Table 1, main text), while the remaining 4 conformers had remarkably higher energies (ΔE > 2.2 kcal/mol compared to the lowest-energy conformer) and were not considered further.

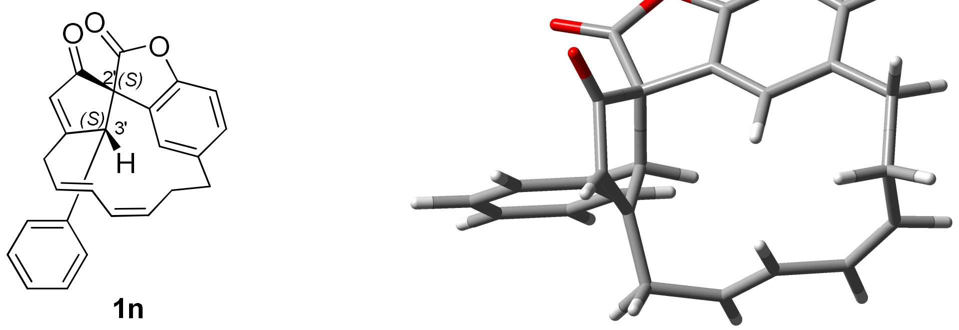

Figure S1. The simplified model compound **2n** for the northern diarylheptanoid unit of unit of zosterabisphenone C and its lowest-energy conformation at the B3LYP/6-31+G(d,p) level.

The favored conformation of the southern diarylheptanoid unit, which is identical for all zosterabisphenones, was known from our previous study[3] and confirmed by ROESY data (Figure 1 and Table 1 in the main text).
Therefore, the two possible diastereomers of zosterabisphenone C were generated by replacing the phenyl group of **1n** with either enantiomer of the southern unit, to give **1** (the 9*R*,2'*S*,3'*S* stereoisomer) and *epi*-**1** (the 9*S*,2'*S*,3'*S* stereoisomer). The torsion angle around the C-8/C-3' bond was unknown, so a conformational search for this degree of freedom was performed using a relaxed energy surface scan in Gaussian 16 (ModRedundant keyword). The dihedral angle C-9/C-8/C-3'/C-2' was incremented in steps of 10° and kept fixed while the geometry of the rest of the molecule was optimized at the B3LYP/6-31G(d) level. The resulting graph of the potential energy vs. dihedral angle (Figure S2) showed one low energy conformer for **1** and two low-energy conformers for *epi*-**1**.
Finally, the conformers of **1** and *epi*-**1** were reoptimized at the B3LYP/6-31+G(d,p) level, and the resulting geometries (Tables S1 and S2) were used for NMR (Tables S3-S5, Figure S3) and ECD (Table S6, Figure S12) prediction. The results are discussed in the main text.

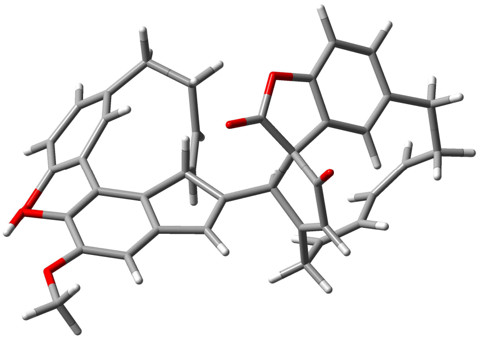

1, conformer 1

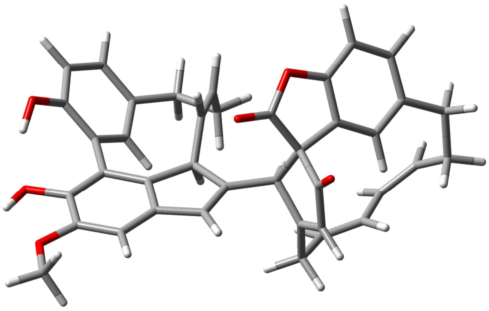

epi-**1**, conformer 1

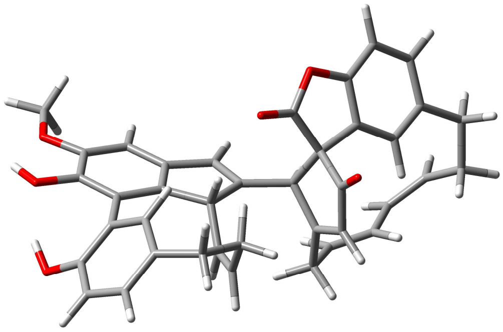

epi-**1**, conformer 2

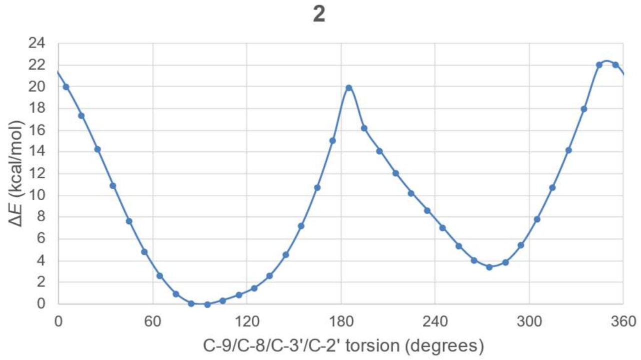

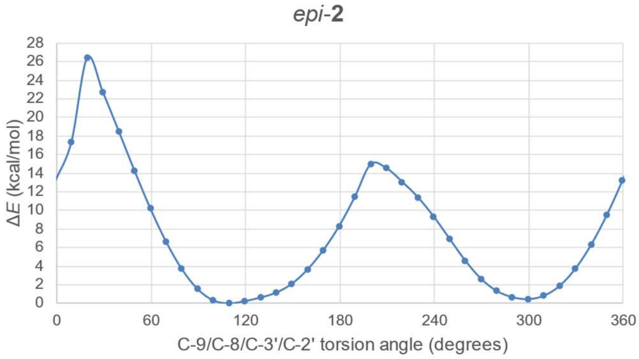

Figure S2. DFT energy of zosterabisphenone C (**1**) and its C-9 epimer *epi*-**1** as a function of the torsion angle about the C-9/C-8/C-3'/C-2' bond, i.e. the bond that connects the two cyclic diarylheptanoid units. The significantly populated minimum energy conformations are shown above the graphs.

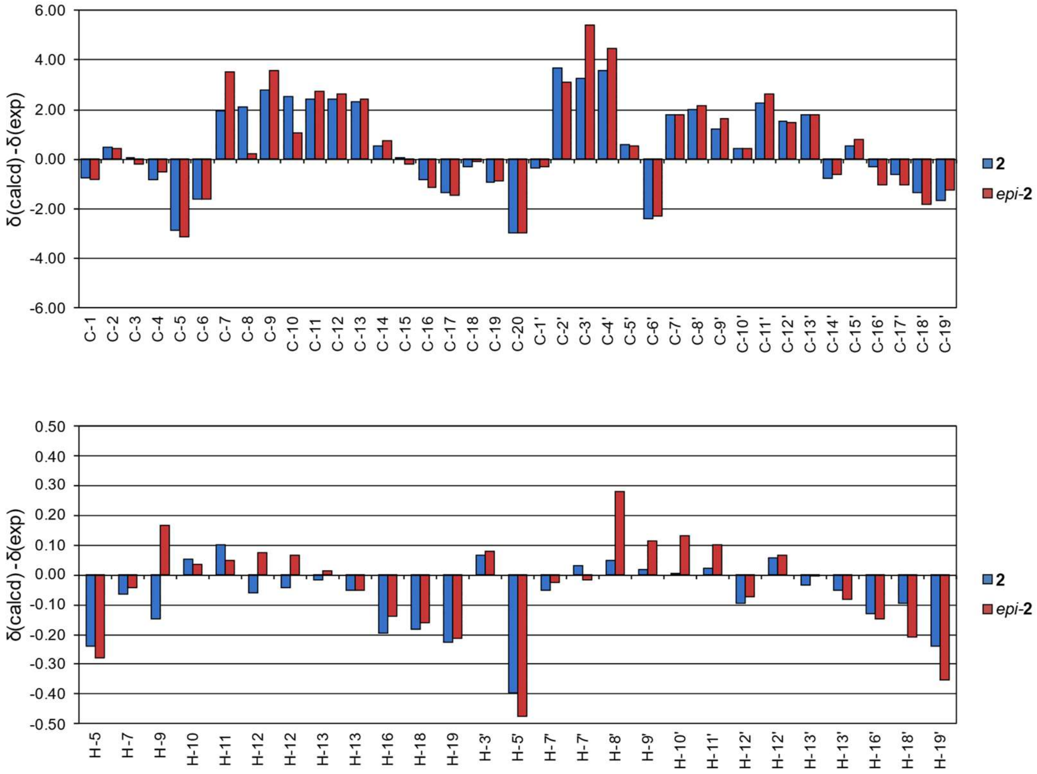

Figure S3. Deviations from experimental values of calculated ^{13}C (top panel) and ^{1}H (bottom panel) NMR chemical shifts of zosterabisphenone C (**1**) and its C-9 epimer *epi*-**1**.

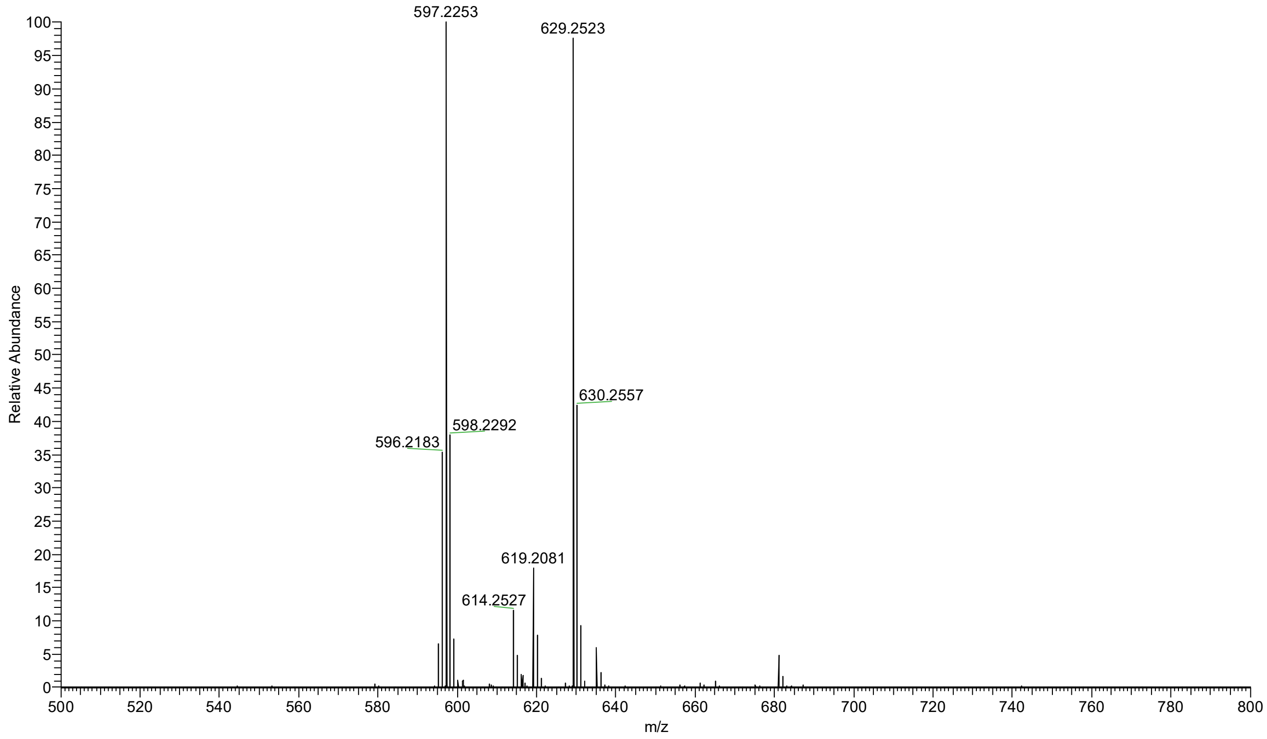

Figure S4. High-resolution ESI mass spectrum of zosterabisphenone C (**1**) (positive ion mode, MeOH/0.1% aqueous HCOOH).

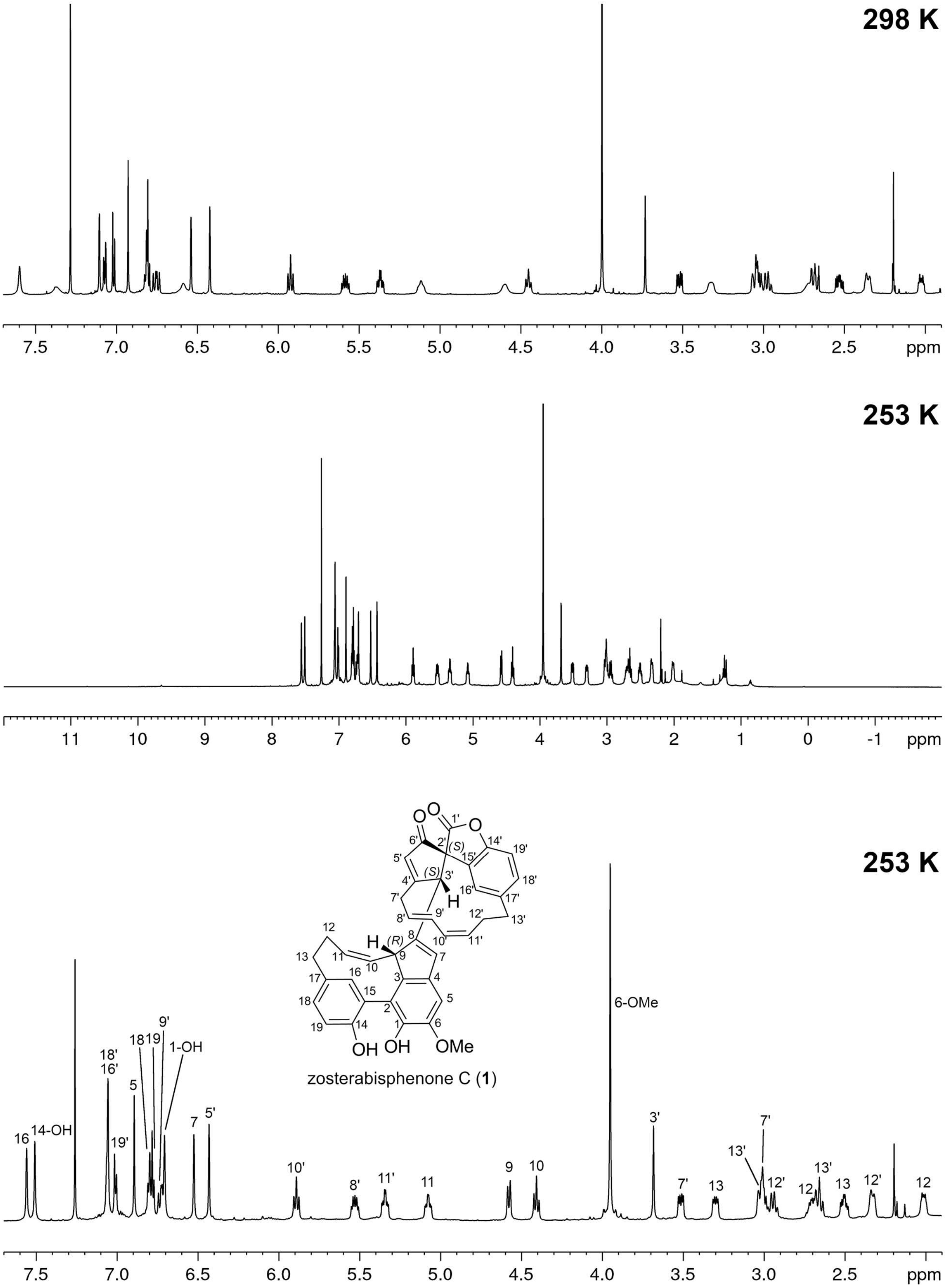

Figure S5. ^{1}H NMR spectrum of zosterabisphenone C (**1**) recorded at 298 K and 253 K (700 MHz, $CDCl_3$)

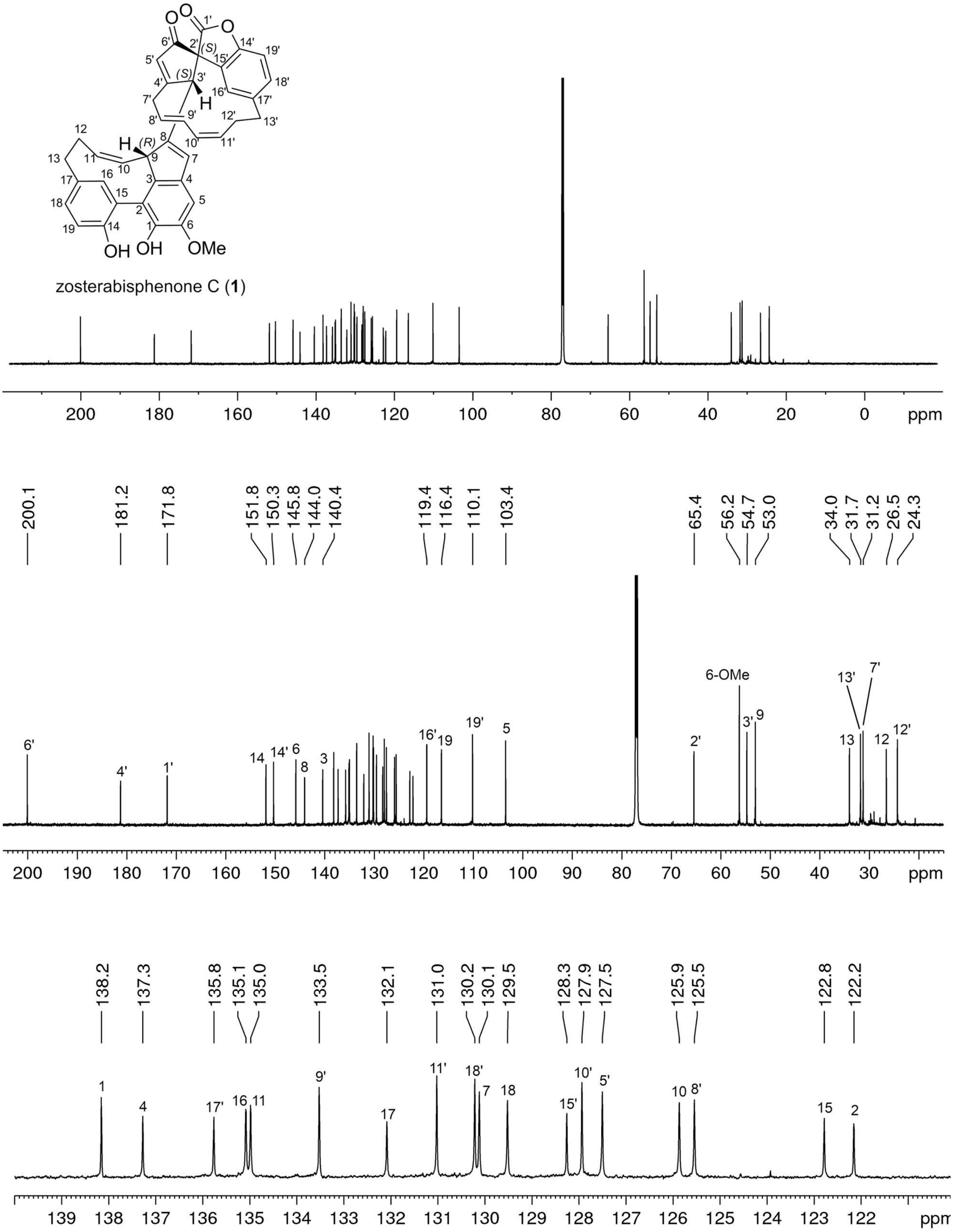

Figure S6. ^{13}C NMR spectrum of zosterabisphenone C (**1**) recorded at 253 K (700 MHz, $CDCl_3$)

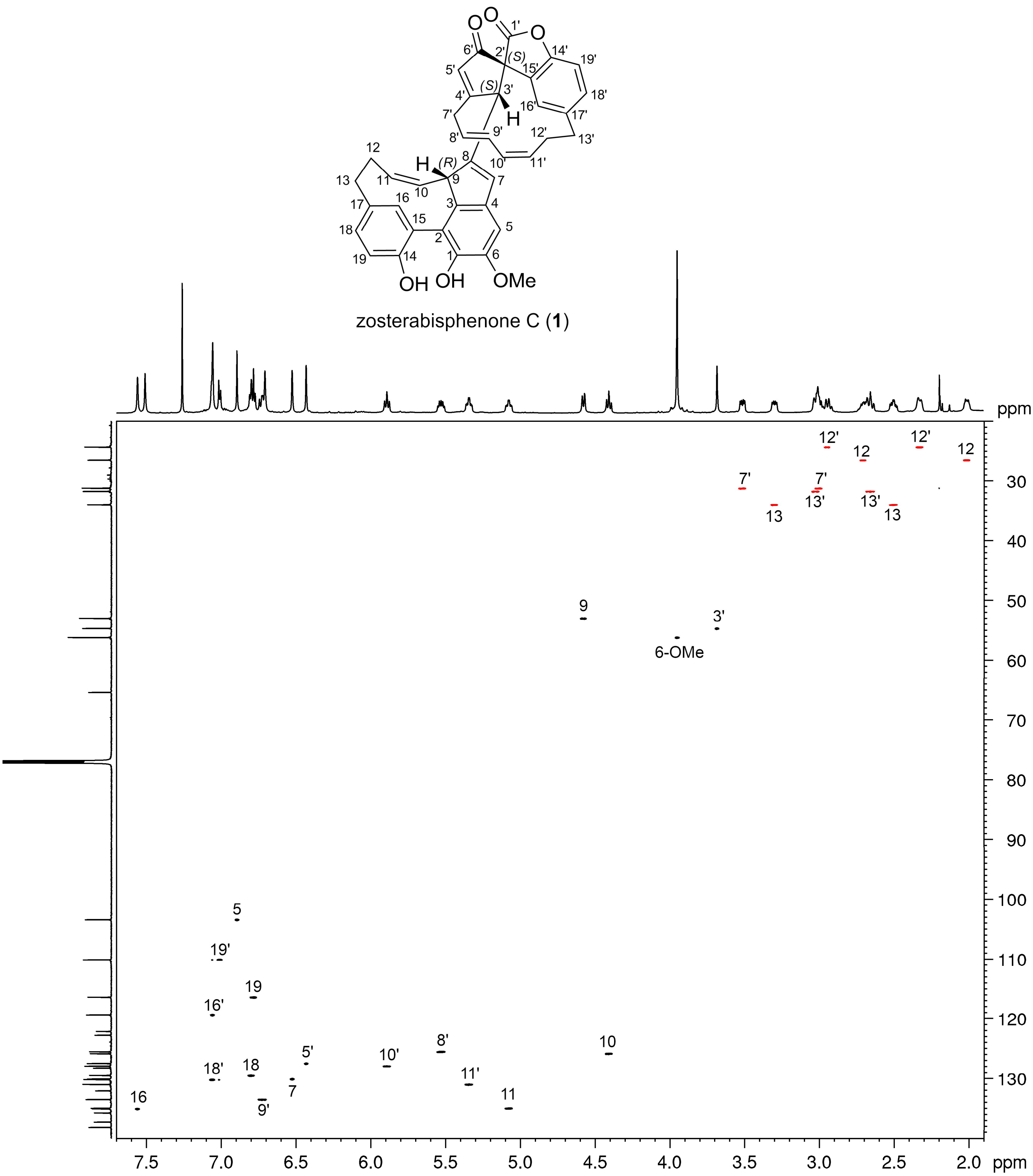

Figure S7. HSQC spectrum of zosterabisphenone C (**1**) recorded at 253 K (700 MHz, $CDCl_3$)

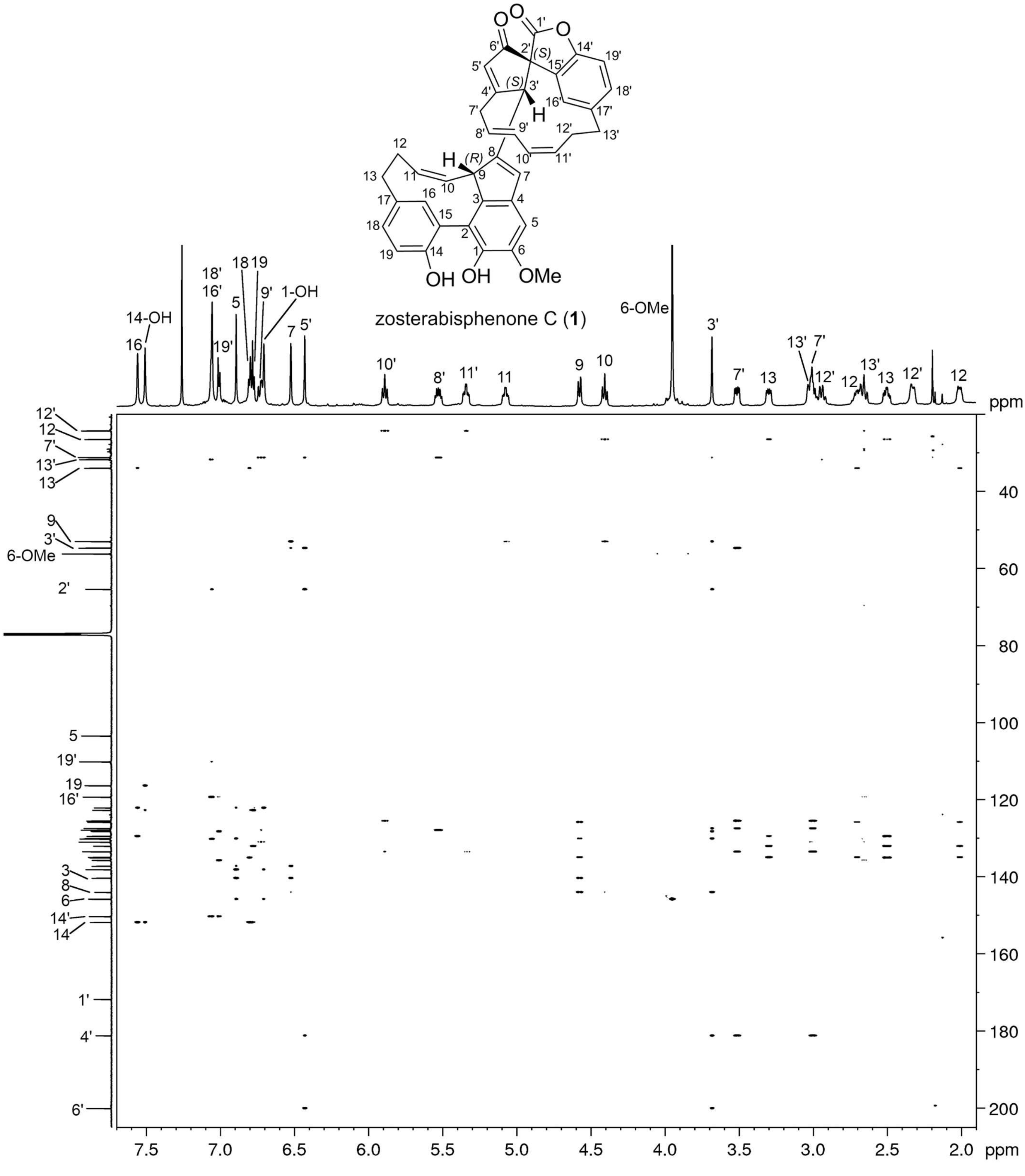

Figure S8. HMBC spectrum of zosterabisphenone C (**1**) recorded at 253 K (700 MHz, $CDCl_3$)

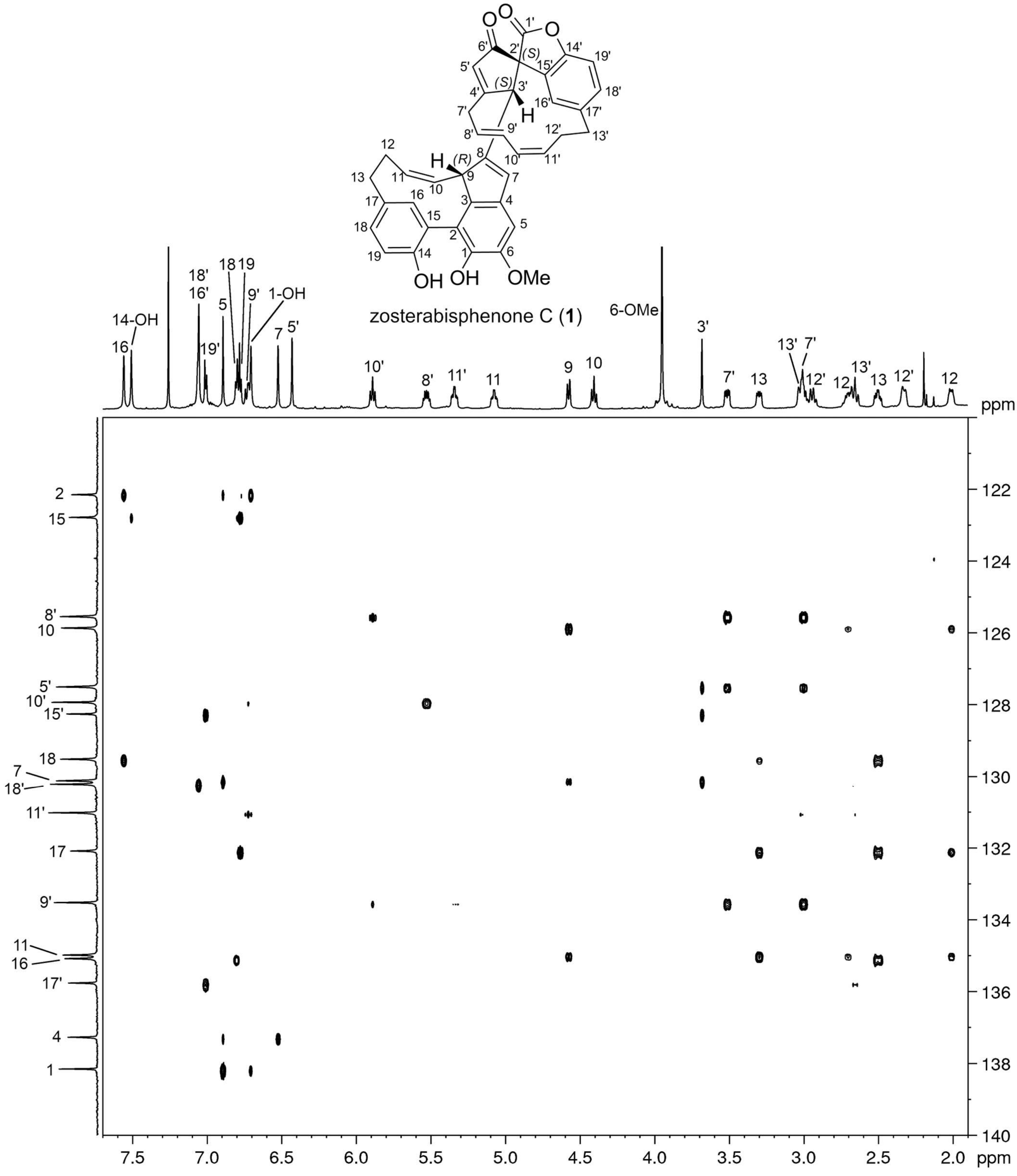

Figure S9. Expansion of the HMBC spectrum of zosterabisphenone C (**1**) recorded at 253 K (700 MHz, $CDCl_3$)

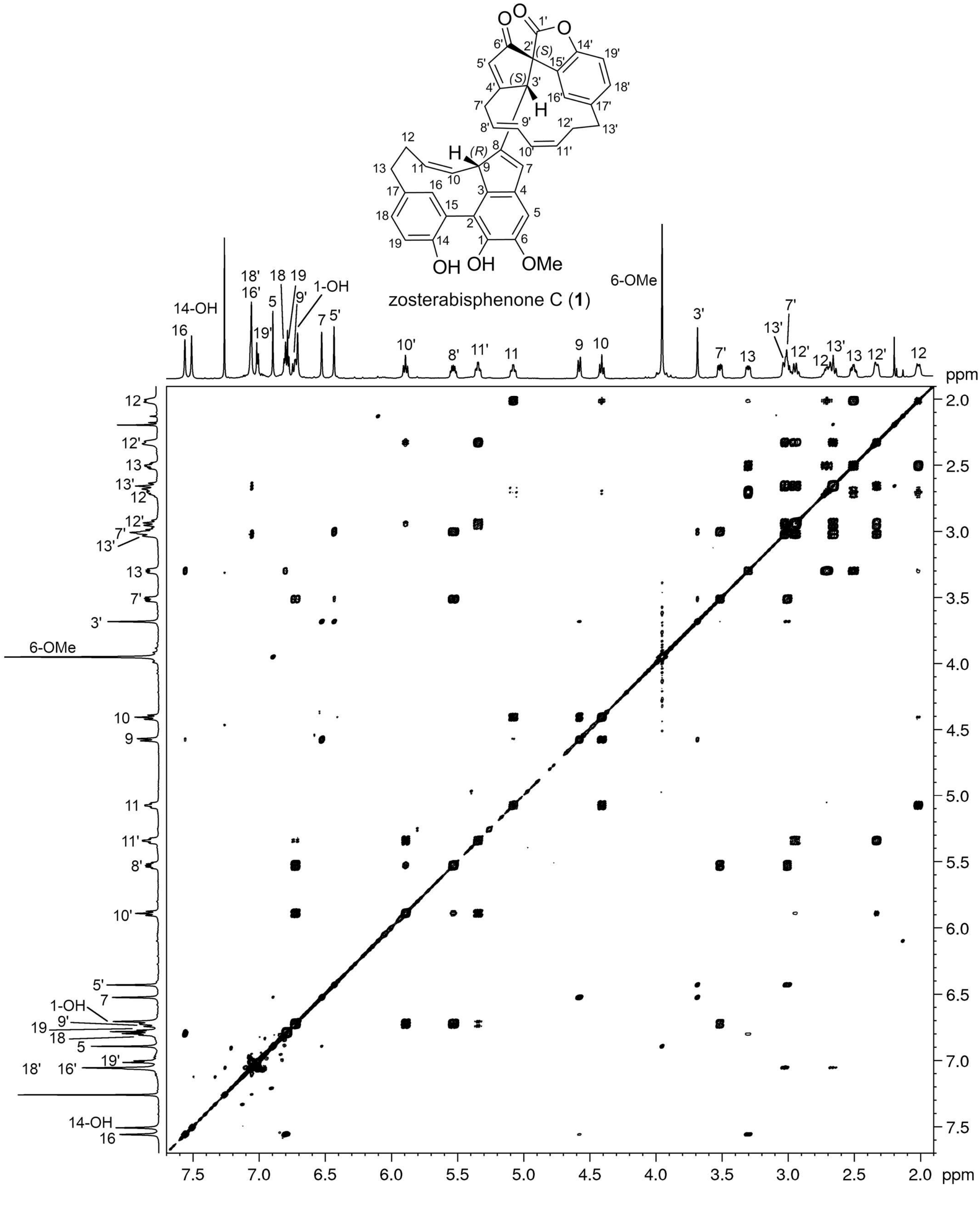

Figure S10. COSY spectrum of zosterabisphenone C (**1**) recorded at 253 K (700 MHz, $CDCl_3$)

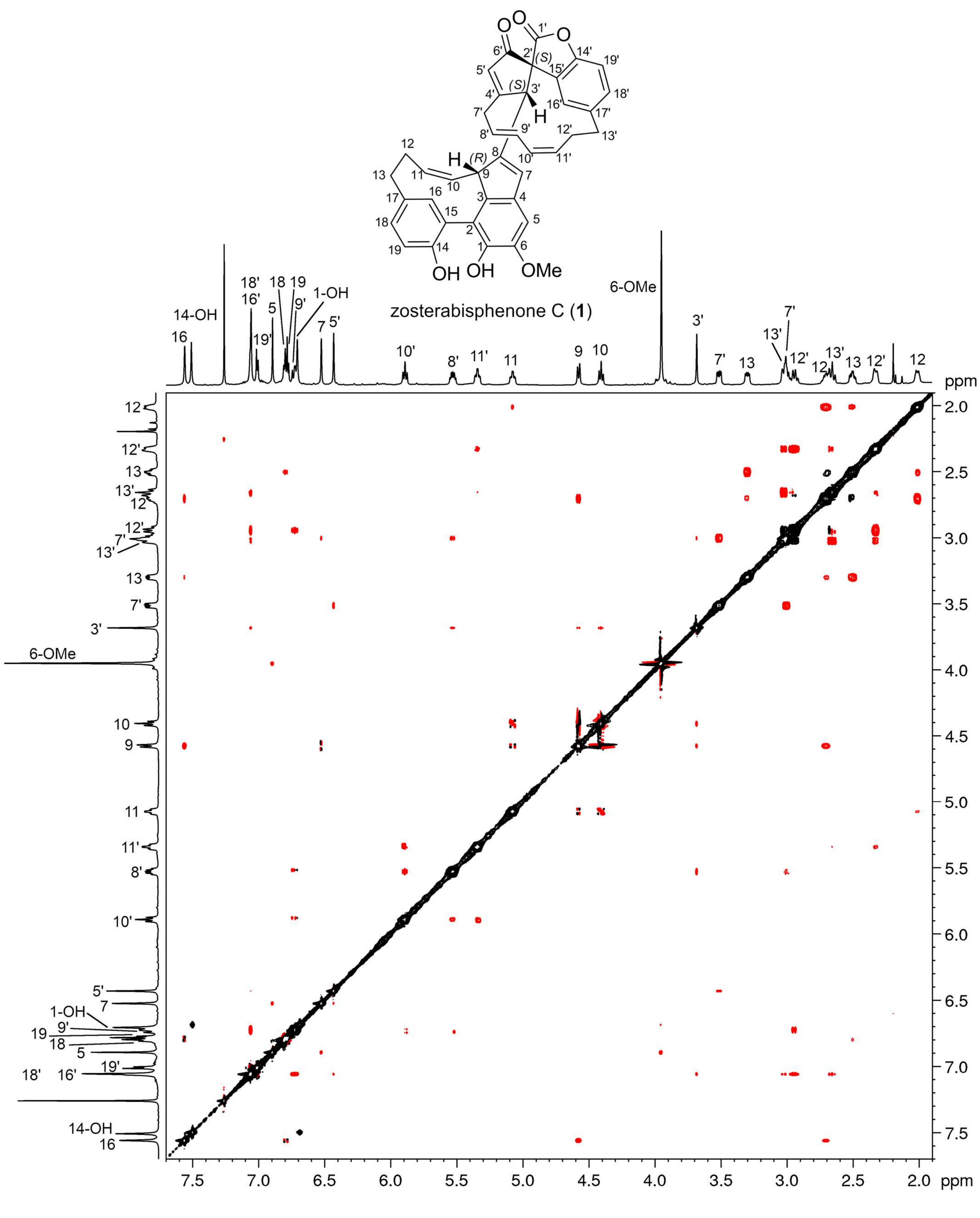

Figure S11. ROESY spectrum of zosterabisphenone C (**1**) recorded at 253 K (700 MHz, $CDCl_3$)

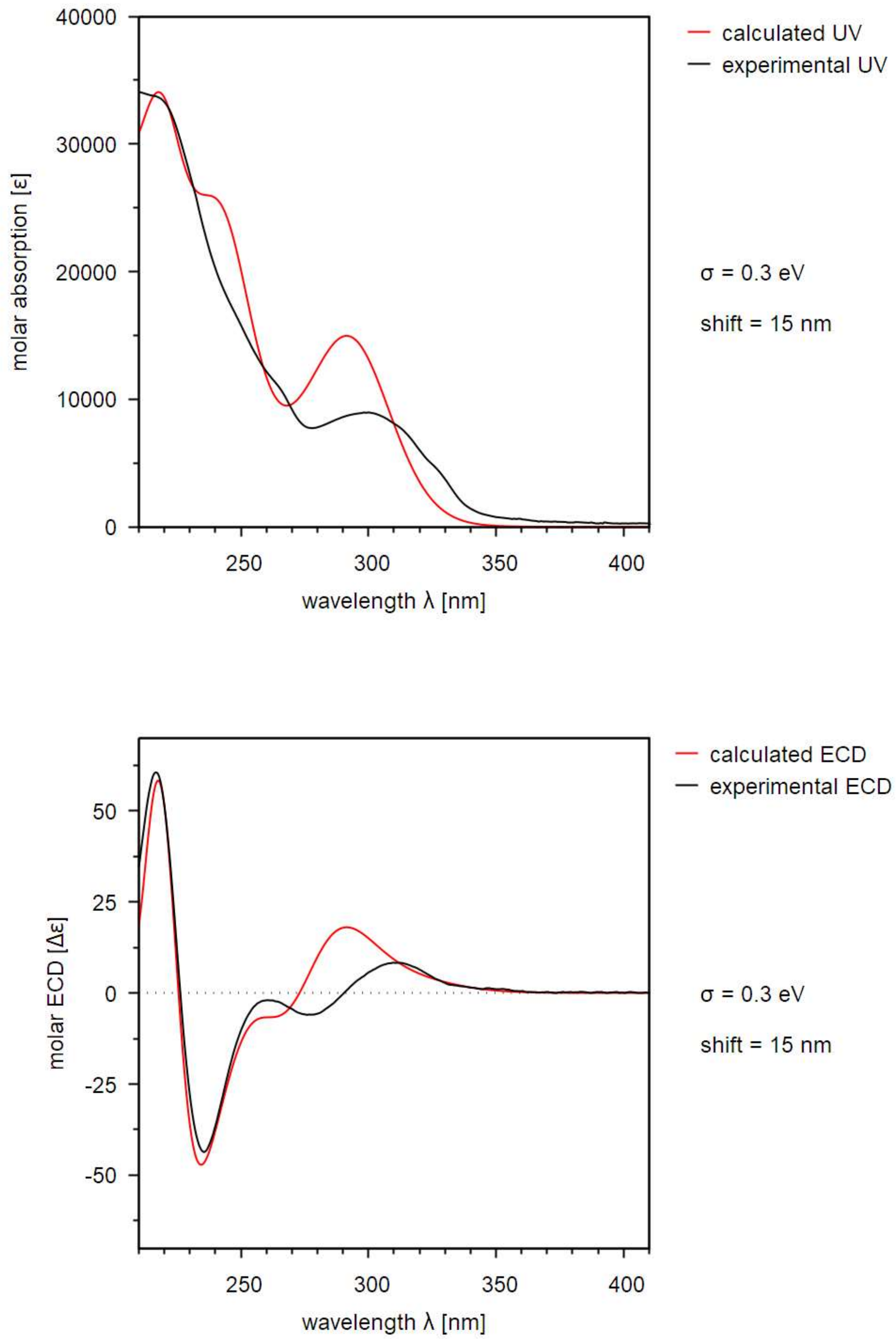

Figure S12. Top panel: calculated and experimental UV spectra of zosterabisphenone C (**1**) in acetonitrile; bottom panel: calculated and experimental ECD spectra of zosterabisphenone C (**1**) in acetonitrile.

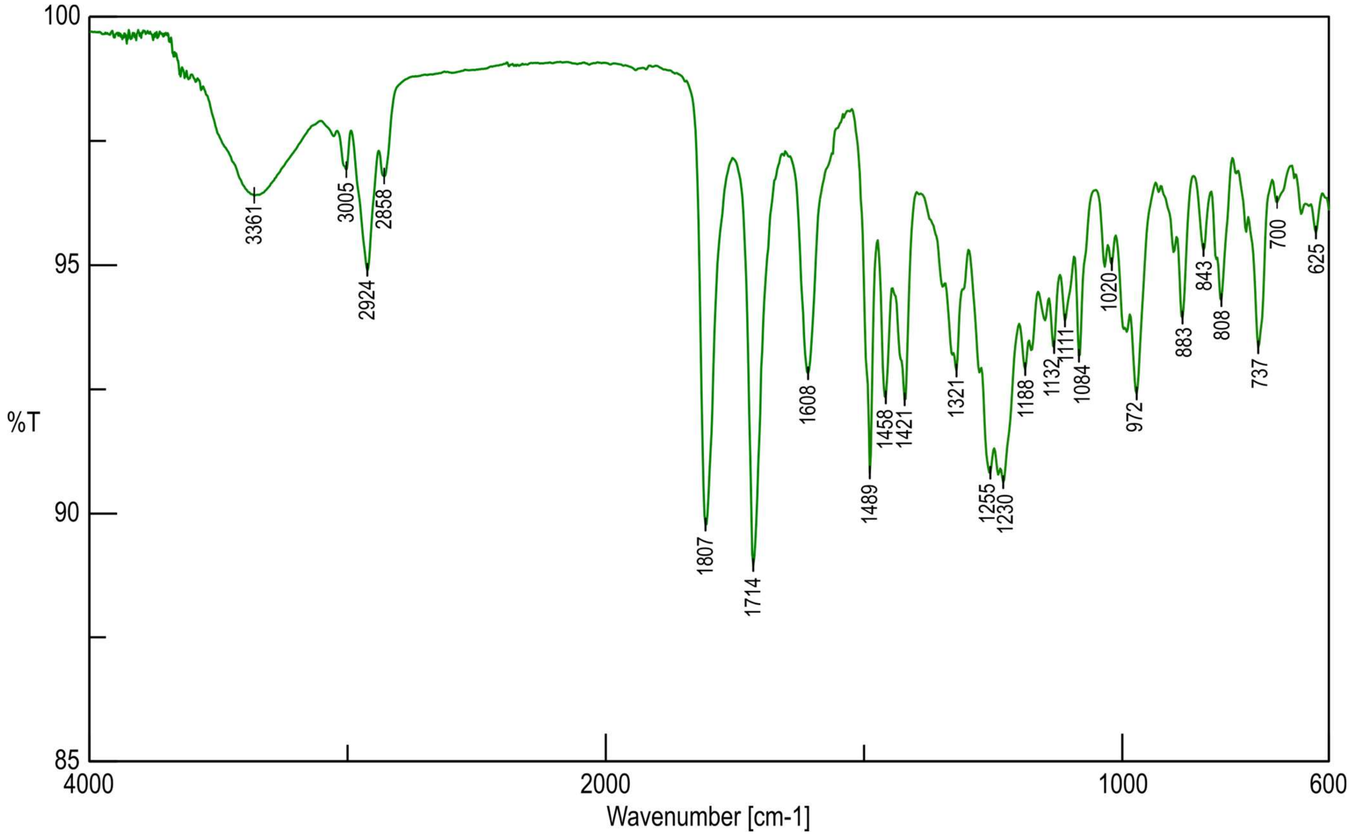

Figure S13. IR spectrum of zosterabisphenone C (**1**) (solid film)

Table S1. Cartesian coordinates of the lowest-energy conformer of zosterabisphenone C (**1**) optimized at the B3LYP/6-31+G(d,p) level.

conformer 1, *E* = -1956.7878077 Harthrees >99% population			
C	4.86191	-0.78656	-0.06881
C	3.76849	0.09374	-0.05490
C	2.48645	-0.50162	0.04945
C	2.35377	-1.89793	0.17152
C	3.45983	-2.75697	0.16403
C	4.71884	-2.18321	0.03475
C	0.94158	-2.24190	0.28924
C	0.18166	-1.12891	0.21438
C	1.06885	0.10289	0.00780
C	0.73993	1.23455	0.96197
C	0.32271	2.47650	0.65752
C	0.18062	3.11477	-0.70597
C	1.31461	4.15034	-1.03143
C	4.77193	2.31901	0.76982
C	3.92352	1.57729	-0.08883
C	2.97607	2.29553	-0.82689
C	2.66870	3.63318	-0.58698
C	3.49800	4.33565	0.29544
C	4.57089	3.69748	0.91726
C	5.88893	-4.28627	0.04776
C	-1.31628	-0.83326	-2.37871
C	-2.08899	-1.23252	-1.11567
C	-1.32505	-1.03058	0.25962
C	-1.92672	-2.13710	1.12009
C	-2.50378	-3.09943	0.37241
C	-2.50791	-2.73419	-1.05487
C	-1.88009	-2.05928	2.62758
C	-2.55511	-0.77671	3.09464
C	-3.86527	-0.52703	2.91673
C	-4.48878	0.77709	3.11332
C	-5.63782	1.20313	2.54813
C	-6.56782	0.44336	1.63712
C	-6.61792	0.97824	0.18164
C	-3.12888	0.50842	-2.30164
C	-3.30191	-0.33377	-1.21077
C	-4.42894	-0.20443	-0.40867
C	-5.36316	0.81077	-0.66536
C	-5.13925	1.65851	-1.76494
C	-4.02922	1.51597	-2.60988
H	3.32898	-3.82940	0.24950
H	0.57438	-3.25763	0.39239
H	0.88107	0.43539	-1.02080
H	0.84513	0.97991	2.01658
H	0.12910	3.14286	1.49907
H	-0.78526	3.63218	-0.76979
H	0.16469	2.35069	-1.48895
H	1.10859	5.09565	-0.51604
H	1.27589	4.36526	-2.10744
H	-4.57051	-0.88370	0.41939
H	2.36133	1.73873	-1.52232
H	-5.85343	2.45077	-1.97538
H	3.29239	5.37929	0.52277
H	-3.87421	2.17000	-3.46100
H	5.23135	4.23626	1.58932
H	-1.58958	-0.04708	0.66039
H	-2.95015	-4.02046	0.73128
H	-2.37079	-2.94330	3.05016
H	-0.83819	-2.07260	2.96602
H	-1.90998	0.01340	3.47583
H	-4.48918	-1.32634	2.51955
H	-3.91956	1.49090	3.70793
H	-5.93002	2.23574	2.74019
H	-7.58719	0.52688	2.03758
H	-6.33926	-0.62676	1.63281
H	-6.88506	2.04230	0.20541
H	-7.45311	0.47334	-0.32414
H	6.75853	-1.02224	-0.19801
H	6.09777	0.96909	1.09570
H	6.92876	-4.60757	-0.01784
H	5.32197	-4.69858	-0.79490
H	5.45606	-4.63578	0.99244
O	6.13846	-0.27445	-0.18933
O	5.91512	-2.86223	-0.00480
O	5.74182	1.75594	1.54854
O	-0.29981	-1.28964	-2.82295
O	-2.85224	-3.40984	-2.00330
O	-1.96295	0.22950	-3.00005

Table S2. Cartesian coordinates of the lowest-energy conformers of the C-9 epimer of zosterabisphenone C (*epi*-**1**) optimized at the B3LYP/6-31+G(d,p) level.

Conformer 1, E = -1956.7858667 Harthrees<br>ΔE = 0.00 kcal/mol, population 75.1%				Conformer 2, E = -1956.784983 Harthrees<br>ΔE = 0.55 kcal/mol, population 24.9%			
C	-0.8087401805	3.4323325795	-3.8001763021	C	-4.382483687	-1.9000824549	0.4303581234
C	-0.7365996568	3.0896488225	-2.4404684911	C	-3.7108803418	-0.6891417801	0.1968336442
C	-0.4063811209	1.7410024612	-2.1524962647	C	-2.3018194796	-0.7705861213	0.0503736753
C	-0.137396124	0.8308571017	-3.1934179075	C	-1.655887671	-2.0204720785	0.0992395821
C	-0.2015256604	1.1998259101	-4.5429277148	C	-2.3474085173	-3.218821403	0.3178346725
C	-0.5453354499	2.5140821401	-4.8344463926	C	-3.7233452932	-3.1424898449	0.4904734868
C	0.1906200139	-0.4725896931	-2.6277894071	C	-0.2255116274	-1.8246225222	-0.0848592334
C	0.1314438002	-0.4234573879	-1.2808572787	C	0.06575218	-0.5119877554	-0.2249334597
C	-0.3029086083	0.9719943198	-0.8212769268	C	-1.2210558186	0.3205481105	-0.1279748819
C	0.5831752081	1.5358402034	0.2732978614	C	-1.4298369774	1.3068229932	-1.2640841456
C	0.2256949998	1.8850964511	1.5220196623	C	-1.547018718	2.6429798042	-1.1786769879
C	-1.1571468112	1.9261741215	2.1326713984	C	-1.5644193191	3.5087147516	0.0609800449
C	-1.728589073	3.3794939088	2.2904384418	C	-2.9846945255	4.0874449513	0.3959588132
C	-0.2748401978	5.3369127273	-1.2616052471	C	-5.5345296383	0.8343996497	-0.7979532323
C	-0.9298798765	4.0826575314	-1.3430346411	C	-4.4282512797	0.6116465143	0.0590511414
C	-1.587713377	3.6329519431	-0.1922628474	C	-3.8052272899	1.7391622275	0.6052203791
C	-1.434976239	4.218806821	1.062280311	C	-4.0567817867	3.0394655404	0.17151808
C	-0.7499179279	5.4383277492	1.1222936536	C	-5.1344234618	3.2305171001	-0.701116037
C	-0.2308963722	6.0148294992	-0.0359639842	C	-5.8977090831	2.1452483369	-1.1309289962
C	-0.381265226	2.2288924263	-7.2187135408	C	-4.0040129889	-5.5059960753	0.845274567
C	2.9535838642	-0.9684459958	-0.3011323956	C	1.417213881	0.548788832	2.2694023929
C	1.8587859814	-2.0178564044	-0.0662544508	C	2.1068772094	0.7598580326	0.9150349915
C	0.3636018673	-1.5455848595	-0.2947844433	C	1.4911945559	-0.0117191588	-0.3288743435
C	-0.3070653573	-2.8317856531	-0.7701391556	C	1.7718803498	0.9618933036	-1.4724884786
C	0.5895476161	-3.7355942052	-1.2145498503	C	2.0997096364	2.1928688033	-1.0346418219
C	1.9690100815	-3.2742228237	-0.9849193153	C	2.1544503296	2.2414210201	0.4333292297
C	-1.8042520019	-3.0044948115	-0.6732740571	C	1.7661693318	0.477900597	-2.9022167516
C	-2.252207878	-2.8349656944	0.7727219691	C	2.7608946344	-0.6652539587	-3.0629017984
C	-1.8594607009	-3.6513235843	1.7678997043	C	4.0832747459	-0.5312344995	-2.8503270027
C	-2.0521377277	-3.373183526	3.1867500587	C	5.0160667585	-1.6473758586	-2.7384430221
C	-1.3319395259	-3.8889048819	4.2051578126	C	6.2157885704	-1.6287546137	-2.1198157101
C	-0.2156089113	-4.9002442241	4.1453459307	C	6.9128713584	-0.4667207955	-1.4585242339
C	1.1793807495	-4.3441182481	4.5358412748	C	7.0563069818	-0.5906006383	0.0817793732
C	3.0946123863	-1.5461076764	1.8746622278	C	3.497518627	-0.2883809932	2.4931080056
C	2.0996658713	-2.3803197079	1.3816659879	C	3.4975026026	0.2442468948	1.2113371998
C	1.482811341	-3.2972006192	2.2231612206	C	4.6421561047	0.1690719697	0.4283578075
C	1.8228089255	-3.3445106242	3.5833914078	C	5.7808401445	-0.4942501665	0.9087632469
C	2.8177397493	-2.4667564727	4.0503384065	C	5.7346692516	-1.0410532271	2.2040997577
C	3.4801182049	-1.5638506951	3.206188075	C	4.6012620666	-0.9374954669	3.0240020123
H	0.0184776758	0.481700107	-5.3241565911	H	-1.8195166503	-4.1646769274	0.3563027642
H	0.4465209753	-1.3422595238	-3.2237142926	H	0.5039561102	-2.6297630298	-0.0696260404
H	-1.3181514899	0.8472443901	-0.4192898213	H	-1.1365903821	0.8713198621	0.8147944346
H	1.6297027584	1.6434895791	-0.0056452467	H	-1.5006976125	0.8534829856	-2.2526785181
H	1.0222548026	2.2600041017	2.1653345538	H	-1.7072490378	3.1764039182	-2.1168315199
H	-1.1351245118	1.4566954989	3.1247180808	H	-0.8712814509	4.3497993628	-0.0685371823
H	-1.8586197889	1.3350173964	1.534758133	H	-1.1956233511	2.9497251983	0.9254035195
H	-2.8032464871	3.3001064958	2.5028405628	H	-2.9629604544	4.4586333385	1.4286973171
H	-1.2731183206	3.8588414741	3.1646255002	H	-3.1934419165	4.9525484747	-0.2441459384
H	-2.1303482084	2.6993026899	-0.26440391	H	-2.9884071662	1.5726523209	1.2958411969
H	-0.5930499801	5.9292372648	2.0801043555	H	-5.3630512632	4.2262450396	-1.0745114768
H	0.2886542477	6.9672242341	-0.0042505203	H	-6.745323592	2.2825223341	-1.7949443043
H	-0.0457277626	-1.2356687281	0.6708304861	H	2.0998610937	-0.9075574722	-0.4850098985
H	0.37239205	-4.7010229258	-1.6584983245	H	2.3044664064	3.0638857842	-1.6471374059
H	-2.0804031919	-3.9911404891	-1.0622524837	H	2.0058357841	1.3136433283	-3.5691729595
H	-2.3021129005	-2.2590845782	-1.3036984412	H	0.7626855075	0.1290270984	-3.1690278556
H	-2.8207625308	-1.9381488399	1.015512872	H	2.3494205764	-1.657702506	-3.2421952846
H	-1.2843875577	-4.5375885545	1.5047050447	H	4.4724451884	0.4681983148	-2.6630239414
H	-2.7952581513	-2.6125648164	3.4246564127	H	4.6596771963	-2.6025205266	-3.1234619112
H	-1.5559849	-3.517087385	5.2052461304	H	6.7546713468	-2.5747559102	-2.0616605302
H	-0.4438033998	-5.7038103647	4.8585699784	H	7.9318371594	-0.4044399975	-1.8638578789
H	-0.1630170543	-5.3828256435	3.1646840839	H	6.4364839787	0.4848956034	-1.7131867862
H	1.1115547914	-3.8872828901	5.5313567284	H	7.5559587053	-1.539254428	0.3156079001
H	1.8536192976	-5.2047364312	4.6494064161	H	7.7476494411	0.1981996599	0.4103318513
H	0.7318650328	-3.9618304938	1.8218577734	H	4.6390665887	0.612121366	-0.5567042491
H	3.091148228	-2.4895466174	5.1024353495	H	6.6087414267	-1.5594205253	2.5904826786
H	4.251883932	-0.898962566	3.5782864739	H	4.582539315	-1.3565397738	4.024073687
H	-1.1439423747	4.7739036983	-5.1251535975	H	-6.0364248351	-2.8011039941	0.7793130806
H	-0.0044969575	5.6376043474	-3.1383641591	H	-6.2479505166	-0.9474377807	-0.8224827619
H	-0.5237717051	2.8588796532	-8.0970941126	H	-4.8392590856	-6.1775281661	1.0459459455
H	0.6534463794	1.8686664347	-7.1877210935	H	-3.5101071162	-5.8036367187	-0.0872499591
H	-1.0695849725	1.3767390082	-7.2624756836	H	-3.2881151392	-5.5511199846	1.6743496609
O	-1.1480465294	4.7231335601	-4.1548868734	O	-5.7507521448	-1.8875438388	0.6138733472
O	-0.6659928503	3.0562674109	-6.0933438188	O	-4.5679372017	-4.202831521	0.7299208391
O	0.4061463122	5.9074135584	-2.296121465	O	-6.2366911751	-0.1672753692	-1.4068177195
O	3.2596293584	-0.4071282445	-1.3152962497	O	0.312916033	0.870167866	2.6157126461
O	3.0051608641	-3.7947356412	-1.34638903	O	2.2949048193	3.2114765422	1.151815932
O	3.6106782635	-0.7102560987	0.8971899887	O	2.2774755988	-0.1121469484	3.1319292309

Table S3. Experimental chemical shifts, calculated isotropic shielding constants, and predicted chemical shifts of zosterabisphenone C (**1**). Calculations were performed at the PBE0/6-311+G(2d,p)/PCM($CHCl_3$)//B3LYP/6-31+G(d,p) level.

	exp. δ_C	shielding constants	calcd. δ_C[a]	$\Delta\delta_C$
C-1	138.2	42.61	137.38	-0.82
C-2	122.2	58.12	122.66	0.46
C-3	140.4	39.42	140.41	0.01
C-4	137.3	43.79	136.26	-1.04
C-5	103.4	81.30	100.65	-2.75
C-6	145.8	35.62	144.01	-1.79
C-7	130.1	48.25	132.02	1.92
C-8	144.0	33.42	146.10	2.10
C-9	53.0	128.56	55.78	2.78
C-10	125.9	52.04	128.43	2.53
C-11	135.0	42.57	137.42	2.42
C-12	26.5	156.84	28.93	2.43
C-13	34.0	149.06	36.31	2.31
C-14	151.8	26.77	152.42	0.62
C-15	122.8	57.96	122.81	0.01
C-16	135.1	45.90	134.25	-0.85
C-17	132.1	49.57	130.77	-1.33
C-18	129.5	52.19	128.28	-1.22
C-19	116.4	65.69	115.47	-0.93
6-OMe	56.2	131.23	53.25	-2.95
C-1'	171.8	6.74	171.44	-0.36
C-2'	65.4	114.54	69.09	3.69
C-3'	54.7	126.26	57.96	3.26
C-4'	181.2	-7.32	184.78	3.58
C-5'	127.5	52.39	128.10	0.60
C-6'	200.1	-20.92	197.70	-2.40
C-7'	31.2	152.55	33.00	1.80
C-8'	125.5	52.92	127.59	2.09
C-9'	133.5	45.32	134.80	1.30
C-10'	127.9	52.05	128.41	0.51
C-11'	131.0	46.86	133.34	2.34
C-12'	24.3	160.13	25.81	1.51
C-13'	31.7	152.02	33.51	1.81
C-14'	150.3	29.82	149.52	-0.78
C-15'	128.3	51.72	128.73	0.43
C-16'	119.4	61.85	119.12	-0.28
C-17'	135.8	44.94	135.17	-0.63
C-18'	130.2	51.61	128.83	-1.37
C-19'	110.1	72.98	108.55	-1.55
			**RMSD ^{13}C**	**1.86**

	exp. δ_H	shielding constants	calcd. δ_H[b]	$\Delta\delta_H$
H-5	6.89	24.46	6.65	-0.24
H-7	6.52	24.68	6.46	-0.06
H-9	4.58	26.90	4.43	-0.15
H-10	4.41	26.86	4.46	0.05
H-11	5.08	26.08	5.18	0.10
H-12	2.01	29.62	1.95	-0.06
H-12	2.70	28.84	2.66	-0.04
H-13	2.50	29.03	2.48	-0.02
H-13	3.30	28.19	3.25	-0.05
H-16	7.56	23.65	7.39	-0.17
H-18	6.80	24.47	6.65	-0.15
H-19	6.78	24.54	6.59	-0.19
H-3'	3.68	27.65	3.75	0.07
H-5'	6.43	25.14	6.03	-0.40
H-7'	3.51	27.97	3.45	-0.06
H-7'	3.00	28.43	3.03	0.03
H-8'	5.53	25.64	5.58	0.05
H-9'	6.72	24.36	6.75	0.03
H-10'	5.89	25.29	5.89	0.00
H-11'	5.34	25.88	5.36	0.02
H-12'	2.33	29.31	2.23	-0.10
H-12'	2.95	28.46	3.01	0.06
H-13'	2.66	28.88	2.62	-0.04
H-13'	3.02	28.50	2.97	-0.05
H-16'	7.06	24.16	6.93	-0.13
H-18'	7.06	24.12	6.96	-0.10
H-19'	7.01	24.33	6.77	-0.24
6-OMe	3.95	27.44<br>27.75<br>27.73	3.75	-0.20
			**RMSD ^{1}H**	**0.134**

[a] ^{13}C chemical shifts were obtained from the isotropic shielding constants according to ref. 9, using the equation: $\delta = (187.3123 - \text{shielding})/1.0533$
[b] ^{1}H chemical shifts were obtained from the isotropic shielding constants according to ref. 9, using the equation: $\delta = (31.7532 - \text{shielding})/1.0958$

Table S4. Experimental chemical shifts, calculated isotropic shielding constants, and predicted chemical shifts of the two conformers of the C-9 epimer of zosterabisphenone C (*epi*-**1**). Calculations were performed at the PBE0/6-311+G(2d,p)/PCM($CHCl_3$)//B3LYP/6-31+G(d,p) level.

		isotropic shieldings				
	exp. δ_C	Conformer 1 ΔE = 0.00 kcal/mol population 75.1%	Conformer 2 ΔE = 0.55 kcal/mol population 24.9%	average	calcd. δ_C[a]	$\Delta\delta_C$
C-1	138.2	42.65	42.65	42.65	137.34	-0.86
C-2	122.2	58.22	57.82	58.12	122.66	0.46
C-3	140.4	40.41	37.36	39.65	140.19	-0.21
C-4	137.3	42.89	45.15	43.45	136.58	-0.72
C-5	103.4	81.44	82.09	81.60	100.36	-3.04
C-6	145.8	35.56	35.95	35.65	143.98	-1.82
C-7	130.1	47.42	43.97	46.56	133.63	3.53
C-8	144.0	35.08	36.39	35.41	144.22	0.22
C-9	53.0	126.98	129.94	127.72	56.58	3.58
C-10	125.9	53.74	53.12	53.59	126.96	1.06
C-11	135.0	42.49	41.42	42.22	137.75	2.75
C-12	26.5	156.98	155.64	156.64	29.12	2.62
C-13	34.0	148.94	148.98	148.95	36.43	2.43
C-14	151.8	26.42	26.97	26.56	152.62	0.82
C-15	122.8	58.22	58.03	58.17	122.61	-0.19
C-16	135.1	46.40	45.58	46.19	133.98	-1.12
C-17	132.1	50.10	48.55	49.71	130.64	-1.46
C-18	129.5	51.87	52.33	51.98	128.48	-1.02
C-19	116.4	65.51	65.93	65.61	115.54	-0.86
6-OMe	56.2	131.24	131.36	131.27	53.21	-2.99
C-1'	171.8	7.05	5.57	6.68	171.49	-0.31
C-2'	65.4	115.62	113.81	115.17	68.49	3.09
C-3'	54.7	125.26	120.26	124.01	60.09	5.39
C-4'	181.2	-8.19	-8.48	-8.26	185.68	4.48
C-5'	127.5	53.21	50.26	52.47	128.02	0.52
C-6'	200.1	-21.29	-20.33	-21.05	197.82	-2.28
C-7'	31.2	152.81	151.72	152.54	33.01	1.81
C-8'	125.5	52.83	52.57	52.76	127.74	2.24
C-9'	133.5	44.82	45.10	44.89	135.22	1.72
C-10'	127.9	52.06	52.03	52.05	128.41	0.51
C-11'	131.0	46.35	46.78	46.46	133.73	2.73
C-12'	24.3	160.12	160.23	160.15	25.79	1.49
C-13'	31.7	152.00	152.06	152.02	33.51	1.81
C-14'	150.3	29.65	29.67	29.65	149.68	-0.62
C-15'	128.3	51.70	50.76	51.46	128.98	0.68
C-16'	119.4	62.57	62.75	62.62	118.39	-1.01
C-17'	135.8	45.58	44.71	45.37	134.76	-1.04
C-18'	130.2	52.11	51.96	52.07	128.40	-1.80
C-19'	110.1	72.43	72.86	72.54	108.97	-1.13
					**RMSD ^{13}C**	**2.10**

	exp. δ_H	Conformer 1	Conformer 2	average	calcd. δ_H[b]	$\Delta\delta_H$
H-5	6.89	24.48	24.60	24.51	6.61	-0.28
H-7	6.52	24.63	24.74	24.66	6.48	-0.04
H-9	4.58	26.82	25.74	26.55	4.74	0.16
H-10	4.41	26.90	26.81	26.88	4.45	0.04
H-11	5.08	26.15	26.08	26.13	5.13	0.05
H-12	2.01	29.51	29.34	29.47	2.08	0.07
H-12	2.70	28.80	28.47	28.72	2.77	0.07
H-13	2.50	29.01	28.95	29.00	2.52	0.02
H-13	3.30	28.23	28.09	28.20	3.25	-0.05
H-16	7.56	23.64	23.44	23.59	7.45	-0.11
H-18	6.80	24.45	24.42	24.45	6.67	-0.13
H-19	6.78	24.53	24.52	24.53	6.60	-0.18
H-3'	3.68	27.75	27.26	27.63	3.76	0.08
H-5'	6.43	25.18	25.39	25.23	5.95	-0.48
H-7'	3.51	27.91	28.06	27.95	3.47	-0.04
H-7'	3.00	28.41	28.70	28.48	2.99	-0.01
H-8'	5.53	25.37	25.43	25.39	5.81	0.28
H-9'	6.72	24.23	24.33	24.25	6.84	0.12
H-10'	5.89	25.14	25.20	25.15	6.02	0.13
H-11'	5.34	25.78	25.81	25.79	5.44	0.10
H-12'	2.33	29.28	29.28	29.28	2.26	-0.07
H-12'	2.95	28.44	28.46	28.45	3.02	0.07
H-13'	2.66	28.85	28.83	28.84	2.65	-0.01
H-13'	3.02	28.54	28.51	28.53	2.94	-0.08
H-16'	7.06	24.18	24.15	24.18	6.91	-0.15
H-18'	7.06	24.27	24.16	24.25	6.85	-0.21
H-19'	7.01	24.48	24.39	24.46	6.66	-0.35
		27.44	27.45			
6-OMe	3.95	27.76	27.75	27.65	3.74	-0.21
		27.75	27.79			
					**RMSD ^{1}H**	**0.168**

[a] ^{13}C chemical shifts were obtained from the isotropic shielding constants according to ref. 9, using the equation: δ = (187.3123 − shielding)/1.0533
[b] ^{1}H chemical shifts were obtained from the isotropic shielding constants according to ref. 9, using the equation: δ = (31.7532 − shielding)/1.0958

Table S5. Experimental multiplicity of ^{1}H NMR signals and predicted ^{1}H-^{1}H *J* couplings (Hz) of zosterabisphenone C (**1**). Predicted couplings whose magnitude is smaller than 0.2 Hz are not reported.

southern unit		predicted ^{1}H-^{1}H *J* couplings (Hz)												
		H-5	H-7	H-9	H-10	H-11	H-12 proR	H-12 proS	H-13 proR	H-13 proS	H-16	H-18	H-19	H-3'
Position	δ$_H$, mult (*J* in Hz)													
H-5	6.89, s		-0.3											
H-7	6.52, s	-0.3		-2.2										-0.9
H-9	4.58, br. d (11.1)		-2.2		11.0	-0.8	-0.7				-0.4			-0.5
H-10	4.41, t (11.1)			11.0		11.4	-0.9	-1.7		0.4				
H-11	5.08, ddd (11.8, 11.1, 4.6)			-0.8	11.4		12.2	5.1		-0.5				
H-12 proR	2.70, overlapped			-0.7	-0.9	12.2		-13.5	11.7	7.2				
H-12 proS	2.01, ddd (13.6, 6.1, 4.6)				-1.7	5.1	-13.5		6.0	1.0				
H-13 proR	2.50, ddd (14.4, 12.1, 6.1)						11.7	6.0		-14.3		-0.4		
H-13 proS	3.30, dd (14.4, 6.7)				0.4	-0.5	7.2	1.0	-14.3		-1.2	-1.1	0.6	
H-16	7.56, br. s			-0.4						-1.2		2.0	0.4	
H-18	6.80, br. d (8.0)								-0.4	-1.1	2.0		8.0	
H-19	6.78, d (8.0)									0.6	0.4	8.0		

northern unit		predicted ^{1}H-^{1}H *J* couplings (Hz)														
		H-3'	H-5'	H-7' proR	H-7' proS	H-8'	H-9'	H-10'	H-11'	H-12' proR	H-12' proS	H-13' proR	H-13' proS	H-16'	H-18'	H-19'
Position	δ$_H$, mult (*J* in Hz)															
H-3'	3.68, s		-0.9	-0.4	-0.8											
H-5'	6.43, s	-0.9		-0.4	-1.9											
H-7' proR	3.51, dd (14.4, 6.5)	-0.4	-0.4		-14.2	7.0	-2.0	0.7	-1.1		1.1					
H-7' proS	3.00, overlapped	-0.8	-1.9	-14.2		8.7	-0.3									
H-8'	5.53, ddd (15.7, 8.3, 6.5)			7.0	8.7		16.0	-0.9	1.1		-1.1					
H-9'	6.72, dd (15.7, 10.8)			-2.0	-0.3	16.0		11.2	-1.3	-0.4	0.8			-0.4		
H-10'	5.89, t (10.8)			0.7		-0.9	11.2		11.5	-0.8	-2.3		0.4			
H-11'	5.34, ddd (11.8, 10.8, 4.8)			-1.1		1.1	-1.3	11.5		12.0	5.4	-0.2	-0.2			
H-12' proR	2.95, dddd (14.2,13.2, 11.8, 2.9)						-0.4	-0.8	12.0		-14.2	13.2	2.8	-0.4		
H-12' proS	2.33, m			1.1		-1.1	0.8	-2.3	5.4	-14.2		2.8	4.9			
H-13' proR	2.66, ddd (16.7, 13.2, 2.9)								-0.2	13.2	2.8		-16.7	-0.6	-0.7	0.2
H-13' proR	3.02, overlapped							0.4	-0.2	2.8	4.9	-16.7		-1.4	-1.4	0.6
H-16'	7.06, overlapped						-0.4			-0.4		-0.6	-1.4		1.8	0.5
H-18'	7.06, overlapped											-0.7	-1.4	1.8		8.2
H-19'	7.01, d (8.6)											0.2	0.6	0.5	8.2	

Table S6. Rotatory strengths (length formalism) calculated for zosterabisphenone C (**1**). Calculations were performed at the ωB97XD/6-31+G(d,p)//B3LYP/6-31+G(d,p) level.

Wavelength (nm)	Rotatory strength (10^{-40} erg esu cm Gauss^{-1})	Wavelength (nm)	Rotatory strength (10^{-40} erg esu cm Gauss^{-1})
155.1	-11.824400	175.9	28.147200
155.2	-0.549600	176.0	-37.723400
155.4	-58.492000	176.1	-6.586400
155.5	0.407900	176.8	-0.875400
155.7	-10.351400	177.1	-8.281600
156.0	-3.914100	177.3	41.302600
156.2	11.719200	177.6	-4.430300
156.6	-18.343800	178.2	-141.712300
156.9	6.907300	178.4	-3.944500
157.2	5.809300	178.7	25.953900
157.4	1.446600	179.2	107.632700
157.5	4.993900	179.7	178.839100
157.6	17.903200	180.5	20.428800
157.9	0.886100	180.7	14.690600
158.0	-22.715300	181.2	-20.485600
158.2	-18.245600	181.6	-166.726800
158.6	-4.862300	182.5	-61.378200
158.7	-17.094800	183.1	1.425700
159.4	-22.768600	184.0	-49.186700
159.6	9.065700	184.4	86.901700
159.7	19.937700	184.8	90.135300
160.0	-8.192400	185.6	-45.600000
160.4	-18.206300	186.0	131.392900
160.7	13.402200	186.7	6.265300
160.8	-2.194600	186.9	4.795800
160.9	-8.593000	187.4	-10.217400
161.4	-48.900200	187.6	56.857400
161.5	-9.330500	188.6	17.003700
161.8	-16.234700	189.2	-3.718800
162.2	-67.387200	190.9	0.364000
162.4	-14.694900	191.2	-11.475800
162.9	-10.421500	192.2	-68.441900
163.1	-23.597500	193.2	-44.054300
163.2	-9.406400	193.9	-38.852200
163.4	-4.122900	194.5	-0.320400
163.6	-4.523500	195.1	-55.919100
164.0	-8.353300	195.5	-4.458300
164.3	-16.411900	196.0	-7.330300
164.8	10.372200	196.6	-34.964100
165.3	51.086300	198.0	58.268000
165.6	38.202900	199.4	28.486300
166.2	-5.377800	200.1	69.846900
166.3	-67.985600	200.5	35.995400
166.4	6.629000	201.8	-56.860600
166.6	9.726500	202.4	-53.673400
167.0	-39.385300	204.8	216.230500
167.3	-30.166300	205.1	311.004800
167.4	2.613600	207.1	-119.681400
167.5	47.430400	209.4	-61.978100
167.6	79.768200	210.8	-31.345700
167.7	-19.769800	211.9	-50.502400
168.6	21.649400	212.6	-3.900800
168.8	-61.798300	213.9	-137.694400
169.0	17.982200	217.0	8.910600
169.2	4.062100	217.3	-28.152000
169.6	-89.753000	218.9	60.494300
169.9	-6.852100	220.6	-84.518700
170.1	59.244800	227.3	56.223600
170.4	-11.999800	227.5	-40.834000
170.5	-7.473500	233.1	-207.239400
170.8	-39.386200	235.2	136.177500
171.5	-69.614900	237.2	23.257900
171.8	-4.079800	239.7	29.133300
172.3	36.183800	250.6	-74.630300
173.2	-83.347900	258.2	9.080400
173.6	102.615500	269.0	-73.029000
173.9	28.350900	270.4	128.473100
174.1	-19.464000	275.0	33.057700
174.7	-0.836700	282.6	8.636000
175.6	-60.436800	303.2	17.373700

Table S7. Effects of zosterabisphenone A (**4**), B (**5**), and C (**1**) on the viability of HCT116 and Hep G2 cells after 48 hours of treatment. The results are expressed as percentage of cell viability. Each value represents the mean ± SEM of 3 experiments including 5–6 replicates for each treatment. Viability of 20% DMSO, used as positive control, was 13.32±0.90 for HCT116 and 1.65±0.32 for Hep G2. ** P < 0.0001

concentration [μM]	zosterabisphenone A (**4**)[a]		zosterabisphenone B (**5**)[a]		zosterabisphenone C (**1**)	
	HCT116	Hep G2	HCT116	Hep G2	HCT116	Hep G2
Ctrl	100.0±1.5	100.0±3.0	100.0±3.0	100.0±2.9	100.0±1.7	100.0±2.2
0.1	97.6±1.5	103.0±4.0	102.0±2.8	97.4±3.4	97.5±1.6	101.0±2.6
0.3	95.2±1.8	112.0±4.5	79.4±3.3 **	91.7±2.0	96.0±1.4	97.6±2.4
1	96.0±1.6	105.0±2.8	76.0±3.7 **	91.6±3.7	95.6±0.9	97.0±2.7
3	95.3±2.1	114.0±3.6	42.9±1.7 **	89.0±4.3	95.5±1.2	105.0±2.4
10	77.3±1.4 **	111.0±2.4	2.6±0.7 **	61.2±6.2 **	27.1±1.2 **	106.0±3.6

[a] Data from Ref. **3**

References

(1) Li, Y.; Mangoni, A.; Shulha, O.; Çiçek, S. S.; Zidorn, C. Cyclic Diarylheptanoids Deoxycymodienol and Isotedarene A from Zostera Marina (Zosteraceae). *Tetrahedron Lett.* **2019**, *60* (32), 150930. https://doi.org/10.1016/j.tetlet.2019.07.021

(2) Grauso, L.; Li, Y.; Scarpato, S.; Shulha, O.; Rárová, L.; Strnad, M.; Teta, R.; Mangoni, A.; Zidorn, C. Structure and Conformation of Zosteraphenols, Tetracyclic Diarylheptanoids from the Seagrass Zostera Marina: An NMR and DFT Study. *Org. Lett.* **2020**, *22* (1), 78–82. https://doi.org/10.1021/acs.orglett.9b03964

(3) Li, Y.; Grauso, L.; Scarpato, S.; Cacciola, N. A.; Borrelli, F.; Zidorn, C.; Mangoni, A. Stable Catechol Keto Tautomers in Cytotoxic Heterodimeric Cyclic Diarylheptanoids from the Seagrass Zostera Marina. *Org. Lett.* **2021**, *23* (18), 7134–7138. https://doi.org/10.1021/acs.orglett.1c02537

(4) Li, Y.; Mangoni, A.; Zidorn, C. Seasonal Variation of Diarylheptanoids in Zostera Marina (Zosteraceae) from the Baltic Sea, Impact of Drying on Diarylheptanoids and Phenolics, and First Report of 3-Keto-Steroids. *Biochem. Syst. Ecol.* **2022**, *103*, 104446. https://doi.org/10.1016/j.bse.2022.104446

(5) Bally, T.; Rablen, P. R. Quantum-Chemical Simulation of 1H NMR Spectra. 2. Comparison of DFT-Based Procedures for Computing Proton–Proton Coupling Constants in Organic Molecules. *J. Org. Chem.* **2011**, *76* (12), 4818–4830. https://doi.org/10.1021/jo200513q

(6) Lodewyk, M. W.; Siebert, M. R.; Tantillo, D. J. Computational Prediction of ^{1}H and ^{13}C Chemical Shifts: A Useful Tool for Natural Product, Mechanistic, and Synthetic Organic Chemistry. *Chem. Rev.* **2012**, *112* (3), 1839–1862. https://doi.org/10.1021/cr200106v. Scaling factors are also available at http://cheshirenmr.info

(7) Grimblat, N.; Zanardi, M. M.; Sarotti, A. M. Beyond DP4: An Improved Probability for the Stereochemical Assignment of Isomeric Compounds Using Quantum Chemical Calculations of NMR Shifts. *J. Org. Chem.* **2015**, *80* (24), 12526–12534. https://doi.org/10.1021/acs.joc.5b02396

(8) Bruhn, T.; Schaumlöffel, A.; Hemberger, Y.; Bringmann, G. SpecDis: Quantifying the Comparison of Calculated and Experimental Electronic Circular Dichroism Spectra. *Chirality* **2013**, *25* (4), 243–249. https://doi.org/10.1002/chir.22138

(9) Edwards, J. M.; Mangion, M.; Anderson, J. B.; Rapposch, M.; Hite, G. Lachnanthospirone, a Dimeric 9-Phenylphenalenone from the Seeds of Ell. *Tetrahedron Lett.* **1979**, *20* (46), 4453–4456. https://doi.org/10.1016/S0040-4039(01)86616-8

(10) Yang, L.; Qin, L. H.; Bligh, S. W. A.; Bashall, A.; Zhang, C. F.; Zhang, M.; Wang, Z. T.; Xu, L. S. A New Phenanthrene with a Spirolactone from Dendrobium Chrysanthum and Its Anti-Inflammatory Activities. *Bioorganic Med. Chem.* **2006**, *14* (10), 3496–3501. https://doi.org/10.1016/j.bmc.2006.01.004

(11) Katsuki, N.; Isshiki, S.; Fukatsu, D.; Okamura, J.; Kuramochi, K.; Kawabata, T.; Tsubaki, K. Total Synthesis of Dendrochrysanene through a Frame Rearrangement. *J. Org. Chem.* **2017**, *82* (21), 11573–11584. https://doi.org/10.1021/acs.joc.7b02223

(12) Sue, D.; Kawabata, T.; Sasamori, T.; Tokitoh, N.; Tsubaki, K. Synthesis of Spiro Compounds through Tandem Oxidative Coupling and a Framework Rearrangement Reaction. *Org. Lett.* **2010**, *12* (2), 256–258. https://doi.org/10.1021/ol902571p

(13) Pérez-Lloréns, J. L.; Acosta, Y.; Brun, F. G. Seafood in Mediterranean Countries: A Culinary Journey through History. *Int. J. Gastron. Food Sci.* **2021**, *26*, 100437. https://doi.org/10.1016/j.ijgfs.2021.100437
